# Perceptions of Health Care Professionals on the Integration and Use of AI in Clinical Cancer Care: Interview Study

**DOI:** 10.2196/83240

**Published:** 2026-04-20

**Authors:** Akhona C Khumalo, Ala Sarah Alaqra

**Affiliations:** 1 Information Systems, Karlstad Business School, Karlstad University, Universitetsgatan 2, Karlstad, 65188, Sweden, 46 73 301 5824

**Keywords:** artificial intelligence, cancer care, oncology, clinical workflows, health care professionals, human factors, sociotechnical systems theory, technology acceptance, UTAUT, skill erosion

## Abstract

**Background:**

Artificial intelligence (AI) is increasingly recognized for its potential to transform cancer care. However, much of the existing evidence of its efficacy comes from controlled settings. There remains a need to complement this knowledge with insights into how AI tools are perceived and used in real-world clinical settings, as well as how their use impacts clinical practice.

**Objective:**

This study aimed to explore key factors influencing clinicians’ acceptance of AI tools and examine how AI adoption and use impact clinical workflows in cancer care.

**Methods:**

We used purposive sampling for recruiting oncology-related health care professionals and collected data using web-based semistructured interviews to gather their perceptions. Data were thematically analyzed and interpreted through the lenses of sociotechnical systems theory and the Unified Theory of Acceptance and Use of Technology.

**Results:**

Participants largely accept and perceive AI tools as beneficial to clinical practice. Unified Theory of Acceptance and Use of Technology constructs were reflected in our data as determinants of intention to adopt AI tools. Trust appears as an influential factor in shaping attitudes toward AI tools. Acceptance is found to both precede AI tool use and to grow following successful integration. The use of AI tools is perceived to yield operational benefits, such as reduced workload and time savings, and clinical benefits, such as increased diagnostic reliability and reduced patient recall. Minimal disruption to clinical workflows following integration of AI tools was reported for some cancer screening applications and organ-at-risk segmentation, whereas greater disruption was anticipated for 3D cancer screening. Although accountability and lack of explainability are highlighted in literature as barriers to AI adoption, participants do not view these as significant obstacles in image-based diagnostic contexts. Additionally, negative impacts, such as overreliance on AI and reduced critical review of AI results, arise in association with the use of AI tools.

**Conclusions:**

Participants perceive AI tools to deliver benefits to clinical cancer care. However, their adoption relies on their alignment with clinical needs and seamless integration into clinical workflows. To encourage clinician acceptance, the identified concerns must be addressed. Future work should focus on training programs, co-design with clinicians, and exploration of mitigation strategies for emerging adverse effects, such as automation bias and potential skill erosion.

## Introduction

### Background

Amid the staggering number of cancer incidences worldwide, artificial intelligence (AI) promises a better future, improving efficacy through faster and more accurate health care decisions [1,2]. This promise can make a huge difference in oncology, where the fight against cancer places a high demand on health care resources, while also being challenged by a workforce shortage. AI is increasingly integrated into various stages of cancer care, including tumor detection, diagnosis, clinical decision-making, risk stratification and prognosis, treatment planning, drug response prediction, and clinical research [3,4]. Although AI has transformative potential in health care and an increasing number of AI products on the market, the number of clinically implemented AI tools is still relatively low [5]. In oncology, evidence of AI’s clinical impact is still limited [4], and there is a lack of understanding regarding how AI is perceived, accepted, and used by clinicians in real-world settings. Juluru et al [6] draw attention to the difficulty of deploying AI tools into the workflow of clinical practice, which poses a challenge to their adoption. While different workflow models have been evaluated in controlled settings, Verma et al [7] highlight that a clear gap exists between research and clinical environments, emphasizing the need for more focus on the role and impact of AI in clinical settings. To bridge this gap, empirical studies are needed to investigate the integration of AI tools into clinical workflows [8] and verify the benefits of AI tool use in the real world [9].

AI can be defined as the ability of machines to perform cognitive functions usually associated with the human mind, such as perceiving, reasoning, learning, interacting with the environment, problem-solving, decision-making, and even demonstrating creativity [10]. These qualities set AI technologies apart and present unique challenges and opportunities that influence user acceptance. In this study, AI tools refer to AI systems, primarily, but not exclusively, based on machine learning and deep learning techniques, designed to perform clinical tasks relevant to cancer care. The value of AI tools in practice can only be fully realized if such technologies are integrated into routine clinical workflows [11,12] and if clinicians are willing to accept and use them [13]. As prior research has shown, user acceptance plays an integral part in any technology’s success [14,15]. Acceptance of AI by clinicians is crucial for its successful integration into clinical practice. AI acceptance depends on how clinicians perceive its impact on their work processes and professional environment [13]. Despite the growing body of research, few studies have examined cancer care-related firsthand perspectives. Furthermore, most prior work has emphasized technical performance, leaving a gap in understanding how AI integrates into real-world clinical workflows. Finally, while acceptance models (eg, Unified Theory of Acceptance and Use of Technology [UTAUT]) and sociotechnical perspectives (eg, sociotechnical systems [STS] theory) have been applied separately, their combined use offers a rich account of how technical, social, and organizational factors jointly influence AI adoption.

This study, therefore, explores clinicians’ perceptions of the use of AI in clinical practice. It aims to identify factors that influence clinician acceptance and the successful integration of AI into routine workflows. We ask the following research questions:

1. What are the perceptions of health care professionals on the integration and use of AI in oncology clinical practice?
  - How does the adoption and use of AI tools impact the clinical workflow in cancer care settings?
  - What factors influence clinician acceptance of AI tools in cancer care settings?

We draw from the STS theory and the UTAUT as lenses for interpreting our data. This study contributes an empirically grounded understanding of AI acceptance and integration from the perspective of clinicians. It identifies and consolidates key factors influencing adoption while highlighting the complex interplay between organizational dynamics, human factors, technological characteristics, and environmental factors that shape AI implementation into clinical practice.

### User Acceptance in AI Adoption

The goal of technology adoption is for the technology to be used to address specific needs, and effective utilization depends on user acceptance. Nonacceptance of technologies by users results in failed technology adoption efforts [16]. Electronic health records (EHRs) offer an example of how health care technologies, if not well accepted or integrated, can negatively affect clinical practice. EHR systems were initially introduced to streamline documentation and improve efficiency, yet many clinicians report that they increase administrative burden, fragment workflows, and contribute to burnout [17,18]. Studies have shown that simple tasks, such as medication renewal, may involve more than 20 steps within the EHR system, requiring clinicians to spend substantial time on manual work [17]. In some cases, physicians spend nearly half their workday on desktop medicine rather than direct patient care, with additional hours spent documenting outside of normal work hours [19]. This slows down workflows and leads to cognitive overload and clinician dissatisfaction [20]. The example with EHRs demonstrates how poorly designed or inadequately integrated technologies can become burdensome rather than supportive.

These concerns also apply to the use of AI in health care. If AI tools are not transparent or explainable, clinicians may be unable to understand or trust the decisions AI tools make, making them reluctant to adopt or rely on such systems [21-23]. This is important in clinical environments where accountability and patient safety are critical. Studies show that the lack of publicly available information on the development, validation, and ethical considerations of approved medical AI tools poses serious transparency gaps that hinder clinicians’ ability to assess their safety and reliability [21]. Similarly, Marey et al [22] indicate how the lack of transparency in complex AI systems, in the case of cardiovascular imaging, poses significant barriers to clinical acceptance. The latter highlights the need for sufficient training of clinicians on AI products [22]. Furthermore, clinician acceptance of AI is strongly influenced by concerns around trust [23]. Clinicians may resist relying on AI tools in their daily practice if such tools introduce uncertainty or misalign with clinical judgment, which eventually results in reducing workflow efficiency and deterring use [13,23].

### A Sociotechnical Perspective

We draw on STS theory to understand AI adoption in oncology. STS emphasizes the interdependence of social subsystems (people and organizations) and technical subsystems (infrastructure, tools, and processes), and the principle of joint optimization: outcomes are best when both subsystems are aligned [24-26]. This lens highlights how AI integration depends not only on technical performance but also on human roles, organizational dynamics, and external influences such as regulations and other environmental factors [26]. Applying STS allows us to examine AI adoption beyond technical efficacy, focusing instead on how AI reshapes clinical workflows and how clinicians, organizations, and technologies co-adapt in practice.

### A Model for User Acceptance

Individual user acceptance has been studied for many years, and many frameworks and models have been developed that effectively explain the factors that influence acceptance and use. In this work, we use the UTAUT. Developed by Venkatesh et al [27], UTAUT synthesized 8 technology acceptance models including technology acceptance model (TAM), theory of planned behavior, motivational model, the combination of TAM-theory of planned behavior, theory of reasoned action, social cognitive theory, the model of PC utilization, and innovation diffusion theory, and proved to have higher explanatory power over all 8. UTAUT has been used in many studies exploring the acceptance of various technologies, including those used within health care. As proposed by Venkatesh et al [27], UTAUT has 4 core constructs—social influence, effort expectancy (EE), performance expectancy (PE), and facilitating conditions—defined as:

- PE refers to the degree to which an individual believes that using the system will help him or her attain gains in job performance [27].
- EE is the degree of ease associated with the use of the system [27].
- Social influence (SI) is the degree to which an individual perceives that important others believe he or she should use the new system [27].
- Facilitating conditions (FC) refer to the degree to which an individual believes that organizational and technical infrastructure exists to support the use of the system [27].

Attitudes, understood as the organization of one’s beliefs about an object, that incline them towards certain actions [28], are also argued as determinants in some literature sources. PE, EE, and social influence are all direct determinants of behavioral intention (BI). BI, in the context of this study, refers to a person’s intent to use AI tools. BI, in turn, determines actual AI tool use. EE, social influence, and PE are moderated by additional constructs: Gender, Age, Experience, and Voluntariness. Experience pertains to the individual’s experience with the new technology. Facilitating conditions, on the other hand, are a direct determinant of actual use [27]. In this study, UTAUT constructs inform our understanding of how AI acceptance takes shape within health care institutions.

## Methods

### Study Design

Given our aim to identify key factors that shape clinician acceptance and the impact of AI integration in clinical practice, this study used a qualitative research design and semistructured interviews to elicit perceptions and opinions on AI use in real-world settings. Semistructured interviews gained popularity for their flexibility and versatility, allowing the researcher to be structured, yet free to explore other relevant ideas that might come up during the interview [29,30]. They are well-suited to research questions aimed at addressing whether a proposed service is warranted**,** how to implement it, how it is performing, or how it can be improved [29]. The interview guide is provided in Multimedia Appendix 1.

We adopted purposive sampling to ensure the inclusion of participants with relevant expertise. The following selection criteria were applied: (1) the person works in oncology or other cancer care-related fields; (2) the work area has implemented, is evaluating AI tools for implementation, or has considered AI tools; (3) the participant is familiar with AI and its uses in cancer-related care. This approach was chosen to specifically target individuals with direct experience or informed perspectives on the use of AI in cancer care. The study included 18 health care professionals involved in cancer care and a medical AI research and development expert. Participants were recruited using professional networks, referrals from prior interviewees, and institutional platforms such as official websites, professional associations, and digital publications. Our sample primarily included professionals using, implementing, or evaluating AI tools for some cancer care-related tasks, as well as those exploring AI tools that could be suitable for use in their clinical areas. A small number of nonusers were also included. Nonusers can offer valuable insights into technology acceptance studies because these participants may elucidate on reasons for nonacceptance. It is also worth noting that some clinicians are involved in research. Table S1 in Multimedia Appendix 2 details the characteristics of the study participants.

### Data Collection

Interviews were conducted online, primarily via Zoom, except for one interview using Microsoft Teams, based on a participant’s preference. Interviews took an average of 42 minutes of discussion ±3 minutes for introductions. Interviews were conducted in English (n=12) and Swedish (n=7). All interview sessions were recorded using an independent recording device (Olympus digital voice recorder) to ensure audio quality and data security. Transcription and translation were carried out using our university-approved (adhering to the university’s policies) tool, which provides initial automated transcription and translation called Amberscript. This was human-checked together with the recording of the interviews to make sure that the content is transcribed properly and translated. This was done by 2 double checkers to make sure of the validity of the data. We have taken additional steps to double-check with some ambiguity regarding the translation of some Swedish-related terminologies. Only one participant was concerned about being misquoted, and the transcript was approved by that participant after reviewing it. Analysis was performed in English.

### Data Analysis

The data were analyzed thematically, following Braun and Clarke’s [31] reflexive thematic analysis (TA). TA is applied to qualitative data to identify, analyze, and interpret patterns of meaning [32]. This is achieved systematically through the iterative process of familiarizing oneself with the data, generating codes from the data, generating initial themes, reviewing the themes, and defining and naming themes. We followed this approach, inductively generating codes and subthemes directly from the data. Since we were using a reflexive TA, saturation was not sought. As discussed by Braun and Clarke [33], the concept of saturation as a rationale for sample size does not align with reflexive TA. In line with their arguments, in our study, the following make it difficult to claim saturation: the semistructured nature of interviews, the lack of homogeneity in our sample, and the inductive nature of our analysis. Instead, we used information power [34] to judge the sufficiency of the collected data. The quality of our dialogues with the specifically purposively sampled group of participants yielded rich data that is relevant to our research aim. The findings align with existing organizational and technology acceptance theories relevant to our research questions. Even though we performed cross-case analysis, we consider the number of participants (n=19) to provide sufficient relevant information, given that implementation and use of AI tools in clinical cancer care is at its early stages and the availability of relevant participants is still sparse.

TA was conducted using NVivo (version 14; Lumivero) and yielded 378 codes. Both authors reviewed the codes and the themes. This involved discussing the codes and deciding their suitability within allocated subthemes and later assessing for fit within the theory-based themes. The inductively generated codes were subsequently organized into higher-order themes aligned with STS theory. In this way, STS theory served as an interpretive lens, not a coding framework. Table S2 in Multimedia Appendix 3 shows how the subthemes map to STS theory components. Although this study does not quantitatively measure acceptance as is commonly done with the technology acceptance model, our results nonetheless align well with UTAUT constructs; hence, the framework was subsequently applied to discuss the results and provide deeper insights into clinician acceptance. The codebook is provided in Multimedia Appendix 4.

### Researcher Reflexivity

The authors do not work in the medical domain. Both authors have a technical background; their knowledge, therefore, informed the development of interview questions and the flow of discussions and influenced the interpretation of results. To mitigate potential bias, the authors iteratively checked codes and themes and held continuous discussions.

### Ethical Considerations

Ethical approval for this study was granted by the ethical adviser of Karlstad University (HS 2023/1591) before data collection. An information sheet and consent form covering both participation and interview recording were sent to participants at least 4 hours before the scheduled interview. At the start of each session, the study was introduced, and participants were allowed to ask questions before proceeding. The consent form is provided in Multimedia Appendix 5. Participants were provided a lunch voucher (≈€20 [US $19.44]) only after the interviews, as a gift in appreciation for their participation. Most of the participants accepted the voucher; however, a few declined.

## Results

### Study Themes

Participants were asked to share their perceptions and opinions on the adoption and use of AI in their professional context, and more broadly in cancer care. Their responses inform 6 themes formed from 28 subthemes. The main themes include General AI Adoption Perceptions to set the stage for the participants’ perspectives on AI, then Social Subsystem (Organization), Social Subsystem (People), Technical Subsystem, Impacts of AI integration as Indicators of Joint Optimization, and External Systems, in line with the components of STS theory. The themes are presented in Table 1, along with the associated subthemes. An extended version of this table that includes the number of coded references and the number of participants linked to each of the subthemes is presented in Table S3 in Multimedia Appendix 6.

**Table 1.** Overview of themes and related subthemes. The letters G, SO, SP, T, J, and E serve as identifiers for the subthemes and their associated theme.

Theme (abbreviation used in subtheme numbering)	Subthemes
1. General AI^a^ Adoption Perceptions (G)	G1. AI^a^ developments G2. Limitations and complexity G3. AI applications
2. Social Subsystem – Organization (SO)	SO1. Organization-specific challenges to adopting AI SO2. Facilitating factors at the organization level
3. Social Subsystem – People (SP)	SP1. Need for AI awareness SP2. Attitudes towards AI SP3. Age-related perceptions SP4. Trust SP5. Involvement in shaping the solution SP6. Responsibility and Accountability SP7. Automation bias SP8. Clinician autonomy, behavioral impacts, and interpersonal factors SP9. Effect on jobs, skills, and competencies
4. Technical Subsystem (T)	T1. AI potential T2. Fit-for-task design T3. Ease of use T4. Challenges relating to data T5. Interpretability and Explainability of AI tools T6. Unsuccessful AI adoption efforts T7. Effects of AI tools on workflows
5. Impacts of AI Integration as indicators of joint optimization (J)	J1. Benefits to clinical practice J2. Clinical benefits J3. Continuity
6. External systems (E)	E1. Regulatory influence E2. Vendor stability E3. AI and clinical guidelines E4. Macro-level enablers

a AI: artificial intelligence.

### General AI Adoption Perceptions

This theme captures the participants’ general perceptions of AI in cancer care.

#### G1: AI Developments

Participants hold varying views about the developments in medical AI. While some perceive it as fast-moving, with a lot happening in a short space of time, others see it as moving more slowly than initially expected. As one participant states:

*I'm a little surprised that we haven't… that we're not further along than we are in those applications. If you think back five years and try to predict where we would be, you would have thought we would have come further.*

[P19]

However, the pervasiveness of AI in society is expected to increase exposure to AI tools, resulting in younger professionals who are more likely to be comfortable with AI use. Similarly, large language models are expected to make an impact in clinical care in the near future. One participant also calls for the health care industry to reconsider its approach and focus more on proactive care. In this area, AI can make a substantial impact, and patients can benefit more in the long term.

#### G2: Limitations and Complexity

A small number of participants express uncertainty about the future of AI and its promise to cancer care. They caution that, in addition to the performance of tools, institutions should also look at the decisions that established the gold standards upon which the tools are built. Furthermore, they argue that the complexity of health care deems AI incapable of handling health issues in the same nuanced way as clinicians. For example,:

*What you realize is that healthcare is so enormously complex… this AI doesn't have human common sense. And a lot of what we do in healthcare is another kind of complex assessment of the best choice for a particular patient, taking into account other diseases, social situation, general status of the patient and the patient’s wishes… there is so much that is weighed into treatment choices.*

[P13]

Currently, AI tools support only a small part of the assessment process.

#### G3: AI Applications

Participants mention several applications of AI, both in use and under consideration, and these include uses in mammography, segmentation of organs at risk, screening for lung and pancreatic cancer, surgery, genetic exams, patient note-taking, patient communication, and data consolidation tools.

### Social System - Organization

#### Overview

This theme examines organizational-level factors that shape AI adoption. Participants discussed both challenges and facilitators to adoption at the organizational level.

#### SO1: Organization-Specific Challenges to Adopting AI

Participants describe the validation and security processes performed at the initial phase as laborious, especially as the same tools must be revalidated in different cities within the same region. They question the logic of repeating these processes. However, they acknowledge that defining boundaries, distinguishing between what is considered local and what is not, and determining whether validation in context is truly applicable to another may be challenging. Participants also highlight the need for continual validation rather than a one-time occurrence. They note the procurement process as another challenge, mainly due to bureaucracy. Participants also identify user buy-in as problematic, with the “staff on the floor,” as one patient puts it, being the hardest to convince that the tools are to their benefit: :

*we have done it this way always. So, why do we need to change how we do things?*

[P1]

Participants also note issues such as a lack of motivation to learn and use new system features, and hesitation to adopt tools that have not been widely accepted, even though there is sufficient evidence to support them. Other reasons include the expectation of big impact solutions before they are worth considering. For example, one participant believes that AI in dermatology will only be truly worthwhile when an AI solution exists that can scan the entire body for suspicious lesions, rather than smaller tools that target a small surface area. Moreover, organizations are said to fail to provide clear guidance on what AI tools to use and how to use them, leaving employees to explore on their own.

Lack of clinician engagement can also be detrimental to AI adoption. In one case, radiologists have been dissatisfied with the implementation of an AI tool. They resist the AI tool because they had not been informed early in the adoption process. This is viewed as continuing to overshadow the perceptions of the involved radiologists, regardless of the same tool having been accepted in other institutions.

#### SO2: Facilitating Factors at the Organizational Level

According to the participants, several factors facilitate the adoption of AI tools at the organizational level. These factors include formal communication, informal social discussions, preparation, and technological orientation of organizational units. Formal communication early on is required to get people on board and reduce skepticism. Participants emphasize the importance of presenting concrete use cases and examples tailored to specific specialties that illustrate how the tools can be beneficial and where they would fit in the workflow. This approach of giving concrete examples is seen as more effective than discussing AI in broad, abstract terms. AI champions are said to play an important role in communication. Informal social discussions among colleagues also play a role in facilitating acceptance. According to participants, the talks in their social circles are concerned with which AI tools to use, which are best at performing specific tasks, and the positive impacts of having introduced AI tools, such as time-saving, which is reportedly very visible to staff.

Thorough preparation before introducing AI tools is another key factor. Participants mention the importance of conducting evaluations and validation and providing sound scientific evidence, characterized by robust methods, sufficiently large datasets, and clear presentation of results, both from internal unpublished reports and publications. According to one patient:

*The most important thing spontaneously is that there is a good scientific basis and a good study with many included patients, where clear results have been shown. That is kind of the basis for being able to get skeptics to believe in AI.*

[P13]

Participants also stress the need to analyze beforehand how implementation would affect people and their daily work. A good approach to preparedness is specifying suitable criteria. A participant from one of the Swedish regions notes that they take into consideration 5 key things: having a good use case, quality of the AI tool, economic viability, being well-integrated technically, and the possibility to validate it within the acquiring health care institution.

The technology orientation of organizational units, along with their tech-savvy experts, appears to be more willing to accept AI tools, though the same can also breed skepticism. Participants note that specialties like radiology and surgery, which are already tech-heavy specialties, may be more open towards trying AI tools, which extend upon what is typically used. Even when the clinicians have negative feedback about the performance of the specific tools they test, there is an impression that they are eager and even excited to try them. Participants highlight that there is ongoing technological development in their fields, and one should be prepared to explore new solutions. However, participants also recognize that not all colleagues are tech-savvy, and those colleagues with less technical experience may still be uncomfortable with the latest technologies. Interestingly, some participants state that while familiarity with technology makes it easier to accept AI, it also, in some ways, makes them more critical and want a deeper understanding of the underlying parts of how the tools work before they can fully accept them.

### Social System - People

This theme explores the perceptions about human and social factors that influence AI acceptance and use, along with the broader impact of AI use on practice and the workforce.

#### SP1: Need for AI Awareness

Discussions with participants underscore the critical need to increase awareness amongst health care professionals about what AI entails, which tools are available for use, how they are used, and the potential unintended consequences that might arise from them. To enable clinicians to detect potential errors emanating from AI tools, participants emphasize the need to enhance professionals’ understanding of how AI tools function, as well as their limitations. One participant stressed that

*You have to know what you are dealing with, of course… you have to know what you are working with to be able to use it in the intended way.*

[P19]

Participants also emphasize that the AI tools can make mistakes and should not be relied upon without critical thinking.

#### SP2: Attitudes Towards AI

Participants communicate that attitudes towards AI tool use are largely positive. They use phrases like “happy we have it,” “generally satisfied,” “very positive,” “healthy and reasonable attitude,” “positive in general,” and some express a desire to work with AI more. Positive attitudes also appear to be influenced by perceived benefits, as well as positive personal experiences with AI tools used for nonclinical purposes. Participants encourage the use of AI tools to support clinical tasks:

*We need to look at it as a helper more, and I think we can work more with the patient if we embrace the AI products that are in development in our different areas.*

[P1]

However, they also stress the need to maintain a critical perspective. To a lesser extent, negative feelings towards AI tools are also evident, emanating from a lack of communication, indifference, skepticism based on their personal beliefs about AI, and fear of the unknown trajectory of AI. Participants note that skepticism diminishes as people begin to see the performance and results of the AI tools.

#### SP3: Age-Related Perceptions

Participants express mixed views regarding age and AI acceptance. Some participants believe that younger professionals know more and have more positive attitudes towards using AI tools. However, a different view also exists, suggesting that there is no consistency regarding whether younger or older generations are more or less likely to accept AI. The participant further explains:

*There are different reasons for different generations to be skeptical or hesitant to use these tools. So, it’s not really the same reason for everyone. Some older people, they just don’t trust this, um, for some younger people, it’s not that much about trust. It’s more about fear or confidence level; and not all of them [reasons] are invalid*.

[P7]

#### SP4: Trust

There are mixed views regarding trust in AI tools. Participants note that the question of trustworthiness of AI tools remains, with concerns about how they can ascertain quality and reliability. Such concerns affect some professionals’ willingness to test AI tools. Nonetheless, others use AI tools for supporting clinical work, even though they highlight that they would not trust them unquestioningly. For example, one says:

*I think that it won’t happen that I blindly trust this. But it will be like a help… like I’m on the right track. It will help me do a potentially better assessment*.

[P8]

Participants also highlight that trust and openness towards AI tools are influenced by previous exposure and experience, scientific evidence, certification of tools, and thorough evaluation in the specific context of use. Some participants question why AI should be trusted less, given that health care professionals already trust and rely on many other technological tools. Still, others point out that health care professionals would rather place greater trust in their own and their colleagues’ abilities over AI tools.

#### SP5: Involvement in Shaping the Solution

The degree to which individuals are involved in shaping AI solutions plays an important role in ensuring alignment with clinical goals. One participant states:

*So, it’s extremely important to work with the companies that have created the AI solution to improve the functionality of the solution. And I have regular meetings with the product developers at that company where they ask questions that they want answers to from a clinical perspective*.

[P2]

Participants describe their involvement as ranging from no involvement to some form of feedback to demonstrations of proposed solutions, when change is required during the validation stage, or when institutions have begun using AI tools. While most participants report limited opportunities for health care professionals to be involved in the design of the AI tools they ultimately use, they emphasize the importance of doing so to reduce problems and anchor the solutions properly to the users’ needs and expectations. They also highlight that, should they have been involved in design, they could have influenced how the tools work to overcome current shortfalls. Health care professionals can give suggestions when the tools fail to fulfill their needs. Participants believe that early engagement greatly influences how users react to the tool’s actual use. They stress that health care professional involvement is necessary during the design stage, and as tools evolve. One participant says:

*I really don’t have specific evidence for what I’m saying now, but it is my experience for many other types of, um, just sort of software tools which clinicians are using. If the clinicians have been involved in the development or early testing of things, they are often quite positive towards the results they get, and they want to use the tools.*

[P4]

However, not all participants agree with this view. A different view is that health care professionals need not be involved early in the design, but rather at a later stage, when the AI solution works at least semi-decently.

#### SP6: Responsibility and Accountability

Although a small number of participants note that it is still unclear who should be responsible and held accountable should something go wrong following an AI tool giving an incorrect result, several participants firmly believe that the responsibility lies with them as clinicians, and they are accountable to the patient. Given that AI tools are intended to support, and the doctors are responsible for verifying their results and making the final decision, the issue of responsibility and accountability does not seem especially problematic. One supported this saying:

*AI is very important, but still needs the radiologists to check their data. So, if an exam goes to the patient with the wrong interpretation, the error is from the radiologist. So, it’s always on the radiologist, not on the AI*.

[P10]

One participant states that it is the developer’s responsibility to build a tool that minimizes the number of false positives, to use sufficiently large datasets, and to carry out performance measurements against established benchmarks. At the operational level, however, many of the participants do not appear to question where responsibility lies.

#### SP7: Automation Bias

A small number of participants have already noted adverse effects of AI use, such as automation bias amongst colleagues. This bias stems from over-reliance on AI tools, either by trusting AI tools more than their judgments or due to finding it so easy to click and move on as opposed to taking time to verify the results. One participant observed that younger professionals are more susceptible to believing and being influenced by AI-generated results. One said:

*When the AI has pointed to something, there’s a tendency among some reviewers to just buy the AI’s decision instead of trusting their own assessment*.

[P14]

Participants stress the importance of exercising caution, verifying results, applying their judgment, and the need for enhanced training to mitigate this problem.

#### SP8: Clinician Autonomy, Behavioral Impacts, and Interpersonal Factors

Participants highlight issues relating to autonomy. As they state, in some cases, clinicians simply resist algorithmic support even when algorithms work. Instead, they would like to feel that it’s their knowledge that gives the correct answer. Regarding the assumption that the clinician determines what is correct, it is also noted that sometimes the AI tool detects cancer that may be difficult for the clinician, or even several clinicians, to spot in an image, thus a patient is assumed to be cancer-free when cancer is in fact present.

Some participants also fear that AI may overtake clinical work, reducing clinicians to passive bystanders and undermining their confidence. A participant emphasizes that it is important for one to acquire one’s skillset first, build knowledge, and gain hands-on experience to trust one’s own judgment. Only then can AI serve as a tool to enhance those skills, which is a far better approach than becoming overly reliant on AI from the outset. They also highlight how dependence on AI tools develops over time. For instance, they may start off skeptical and anxious to use AI tools, but later the opposite may occur, where they become uneasy when the exams have not also been reviewed by the AI tool. In support of this, one states that despite the ability to work without AI tools, a dependency forms as one uses them, and if removed, their absence is noticeably felt. Further to this, another warns that using AI tools daily carries the risk that clinicians will cease to question the results or consider alternative interpretations.

*I guess the fear is always about relying on it too much*.

[P12]

Additionally, participants highlight that the use of AI tools can impact interpersonal relationships. For example, replacing administrative staff may mean they lose support from them, not just for the tasks the AI tools fulfill, but also for the support tasks the administrative staff does in the periphery.

#### SP9: Effect on Jobs, Skills, and Competencies

Participants believe that while there may be a threat to administration staff, clinical staff may not have to worry about job loss for several years. Instead, AI tools are expected to serve as support and complement clinical practice. Although one participant, somewhat agrees to this, they still state a persistent concern regarding job impact:

*But I still think that in the long run we may be working ourselves out, and that it’s just a matter of time. But, it feels like it will take considerably longer than we initially feared.*

[P14]

AI tools handle simple clinical tasks, and the participants stress that the physician’s judgment remains indispensable, both for conducting assessments themselves and for overseeing the AI-assisted processes. Nonetheless, participants perceive a threat to skills that might weaken due to over-reliance on AI tools. Particularly, the younger generation of clinicians who may have more exposure to AI tools may not be getting sufficient hands-on experience. Participants fear that this will result in eroding their confidence in their professional knowledge. To address the issue, they suggest that a plan must be in place that will require a certain amount of work to be done by the clinician, without AI assistance. For example, a radiologist should retain the skill to delineate structures manually. Participants also suggest that younger physicians should redo by themselves the work that AI tools have performed to gain experiential practice. This should be viewed as a necessary part of education. They note that a shift is beginning to happen in medical education towards covering AI fundamentals and preparing medical students for professions that are AI-augmented. In addition, health care institutions provide tool-specific training, helping ensure the correct use of AI tools. Participants regard training to be straightforward and brief, and training sessions typically last about an hour or two. In some cases, multiple sessions are offered. However, attendance in training sessions is also limited by time constraints. In other cases, the AI tools used are deemed intuitive enough to use without prior training.

### Technical Subsystem

This theme covers participants’ perceptions of AI tools in terms of their potential, the effort associated with their use, and the importance of aligning them with specific clinical tasks.

#### T1: AI Potential

Apart from already experienced benefits, participants perceive AI as having significant potential across various areas of cancer care and enhancing their clinical practice. First, participants believe AI tools can help health care professionals in identifying better treatments more quickly; by rapidly collating and analyzing information from various sources, AI can support the selection of the most suitable treatment arm for a specific condition. AI tools can also support compliance with required clinical standards of image taking, particularly for dermatological conditions. In addition, AI tools can aid in evaluating the need for care and improve the safety of medical procedures, as well as relieve radiologists of certain tasks, allowing them more time for other clinical activities. They are also expected to reduce health care costs. One participant explains the potential for better procedures, saying:

*This is with skin cancer. It [AI tool] can help identify and make the correct diagnosis on the right tumors, and that you should operate on the right spot if it is [present], so that you avoid unnecessary excisions. Because even the most experienced dermatologists operate on four benign changes before they find a melanoma*.

[P18]

Another highlighted the potential of AI tools in increasing patient support:

*I think that can also be something where AI can be really helpful, to help us monitor patients throughout their chemotherapy journey*.

[P12]

Second, participants believe AI can aid patients’ understanding of their disease, facilitate understanding of scientific literature about the disease, and help patients feel more in control of their treatment. Thirdly, AI can assist health care professionals in staying up to date with clinical practices and developments. It is also expected to accelerate the translation of research findings into clinical practice.

Even with so much potential, participants point out that these tools still have limitations in handling certain functions that require human judgment. For instance, handling decisions on how to proceed with a patient’s chemotherapy after years of treatment involves deep contextual understanding that only a physician can provide. Furthermore, clinicians are better at handling standardized and systematized nomenclature, an area where AI tools still fall short.

#### T2: Fit-to-Task Design

Participants point to the importance of clear use cases and AI tools that are designed for specific tasks that can support their work. One participant emphasizes that as there are many AI tools on the market,

It is very important that one is very clear on the suitability of the AI tool for its intended use, and are clear on the kind of testing they should do before they can use it.

[P19]

Participants stress that the tools must meet performance expectations and should have clearly defined limits and boundaries. Participants also note that AI tools should help them achieve specific goals such as improving the quality of care, using limited resources efficiently, and improving operational effectiveness. Without this alignment, there is no incentive to adopt such technologies.

#### T3: Ease of Use

Although a few participants note that some AI tools initially require more work, resulting in longer processes, they acknowledged that over time, these solutions eventually show improvement and positive outcomes. The longer processes are associated with the need to change work processes and make adjustments to ensure optimal functioning and fit within the workflow. The participants view this as a natural part of change. One states:

*and there will certainly be a lot of work in the first year when we try to adjust so that it is right and good. So, there will certainly be a period now when things are slower for us and we will not be as productive, but that’s part of it. There is not much to argue about. It’s just that if you believe in it, you have to accept that it will take a while before all the new routines have settled in and become as good and effective as before.*

[P2]

Several of the current AI tools are perceived as easy to understand and easy to use, and where errors occur, recovery requires little effort. One example is that if an AI-based segmentation tool returns incorrectly drawn structures, the radiologists, who possess the expertise to delineate them manually, can easily make corrections. Support is available for health care professionals when using AI tools. The support is provided by vendors, as well as IT departments within health care institutions. High availability and responsiveness when support is needed are appreciated and underscored by phrases like “all the time,” “24/7 almost,” “continuous support,” and “when we have challenging questions... we turn to them.”

#### T4: Challenges Relating to Data

Participants also raise data privacy and security as concerns. Particularly, the potential misuse of patient data sent to the cloud and shared with third-party companies is disconcerting.

*That is one of the three major challenges that we have encountered. It is the technical implementation to get through data protection considerations, patient data considerations, cloud solutions and all that.*

[P9]

However, others point to General Data Protection Regulation compliance and pseudonymization of sensitive data as providing the necessary security. Another ethical concern raised is the potential of AI tools to propagate bias, whether inherent, learned from training data, or intentional. Furthermore, inconsistencies in how colleagues input data can impact AI tool performance.

#### T5: Interpretability and Explainability of AI Tools

To encompass both interpretability and explainability, this section uses the concept of transparency. While most AI tools in clinical practice are admittedly opaque or “black-boxed,” participants express differing views on the necessity of transparency. A lack of transparency is said to introduce uncertainty, as clinicians cannot discern what the model has taken into consideration to reach its conclusions. Some of the participants emphasize the need to understand how the models arrive at their decisions to clarify aspects that may have been missed during a clinical evaluation, or in the case of predictive tools, to support the development of suitable interventions. However, participants also suggest that transparency is more critical in some contexts than others. For instance, with image interpretation, where clinicians can independently verify results by reviewing the same images, transparency may be less critical. On the other hand, some participants feel that they do not need tool transparency. For them, the tool’s high level of performance, paired with supervision by a human expert, is deemed sufficient. For example, one states:

*No, I don’t need to know how it is done inside, how the AI have been calculating. But, of course, I have to compare and see. Do I agree? Is this the right decision? Is it really so suspicious.*

[P19]

Others believe that the level of detail about how a model works would be beyond clinicians’ comprehension. One participant points out that there is a trade-off between accuracy and explainability of AI tools, noting that improvements in one often come at the expense of the other. As a result, they may settle for partial explanations and experiment with different approaches to understand how the models achieve their results. Nonetheless, another believes that transparency of AI tools could offer tangible benefits to clinicians, providing peace of mind, opportunities for learning, and helping them clarify conclusions better for their patients.

#### T6: Unsuccessful AI Adoption Efforts

The following examples provide insight into how AI implementations can fail. Participants shared examples of unsuccessful AI adoption efforts that reveal technical- and implementation-related challenges. In one case, a commercial product, despite having been validated in advance by the purchasing health care institution, resulted in too many false positives once introduced into clinical practice. Another example involves an in-house development project that, when benchmarked against commercial alternatives, performed poorly. Even when technical infrastructure is in place, hurdles remain. One participant describes a locally deployed model as difficult to operate and poorly integrated into the existing pipeline, calling it “a pain” to manage. Custom-built systems are presented as producing minimal gains in comparison to larger commercial solutions. At the same time, the participant cautions that the success attributed to some AI tools, particularly in pathology, is often overstated, with many models still failing to generalize well between patient cohorts. They state:

*So, so far, those claims were, I would say, highly exaggerated because, um, they usually I mean, maybe we haven’t seen the right models, but it’s like they would over analyze each particular sample, like in a particular cohort, it would work well. If you try to generalize it to other cohorts like the slides which we would have, then it would start to behave in a crazy way*.

[P3]

#### T7: Effect of AI Tools on Workflows

Participants note that AI integration changes work routines, but its impact on workflow varies across contexts. Some require more adjustments than others. For example, one participant states that:

*As far as finding breast cancer is concerned, it’s a big challenge to change all our routines so that they work with the new way of examining breasts.*

[P2]

This suggests that clinicians need to accept new ways of working, a process said to be challenging at first and often met with resistance. The participant highlights that workflow changes will be due to the additional steps required in the process of 3D breast cancer screening. Nevertheless, they expect a positive overall outcome eventually. Participants highlight that AI tools in one area of care can significantly alter the patient’s pathway after an examination, and the clinicians must rethink how the quick AI responses will affect operations at different times in the day. In breast cancer screening, for example, the use of AI tools introduces a new process of reviewing images, where an AI tool reviews the images at one point, and radiologists review the images at another. There are different points and scenarios in the workflow where senior or junior radiologists get involved and perform their assessment. One simply puts it:

*The main difference is that some exams go only to one doctor instead of two. So, that’s the only difference in workflow*.

[P17]

Others note that, initially, screening may take a little longer as images contain more information and AI-generated markings that highlight suspicious changes in a breast image. The workflow can also differ depending on the protocols or image modalities used. For instance, whether a computed tomography (CT) scan, magnetic resonance imaging (MRI), or another interface is used. Several participants feel that workflow changes are not drastic and have minimal impact on cognitive load. In one case, however, they note that an AI tool may perform its task satisfactorily, but a lack of integration with other technical systems may cause a workflow disruption. AI integration into the workflow must be well organized and accompanied by staff training to result in a more efficient process. After integrating AI tools in their work, participants note that evidence shows a positive synergy between good AI tools and experienced clinicians.

### Impacts of AI Integration as Indicators of Joint Optimization

This theme reflects the outcome of AI tools and the social system as a jointly optimized system.

#### J1: Benefits to Clinical Practice

Participants note several benefits. The 2 primary benefits that clinicians note are workload reduction and time savings. Workload reduction is highlighted as one of the key goals of adopting AI in clinical settings, which are currently overwhelmed due to staff shortages.

*Well, in Sweden, we have a lack of radiation oncologists. So, that means that we are finally able to do our jobs on time. Otherwise, we were always behind schedule. So now we are on schedule again*.

[P6]

Participants point out that the introduction of AI tools reduces manual work and increases productivity. They explain that in breast cancer screening, AI tools handle scans that would otherwise wait for the radiologist to review. Similarly, in segmentation tasks, the time to draw structures is significantly reduced by the automatic generation of structures compared with when they are done manually. A participant points out that:

*The physicians love it because they say, oh, I’ve got all this time. I don’t need to take this patient [for] three hours. Now I can do something else and take another patient*.

[P16]

In speech recognition systems designed for taking health care records, the need for an administrative middleman is eliminated, resulting in quicker access to health care records for subsequent care. Participants also note that the use of AI results in increased access to information and frees up time, allowing them to work more with patients. One emphasizes that the use of AI tools improves current work processes and adds the ability to perform functions that are otherwise not possible for humans. While they may not fulfill all needs, AI tools are seen as making a difference in the care process. Furthermore, participants highlight that AI tools increase accuracy and reliability, can minimize the risk of oversight, and increase clinician confidence in performing certain tasks. Participants also highlight that in the long run, AI may contribute to reduced costs through paying fewer salaries and reducing the need for multidisciplinary team meetings where expert views of different professionals are corroborated to reach decisions about cancer patients; that is, their diagnosis, recommended treatment, and follow-up care. One participant states:

*And this is a situation where I think this gathering of many different expert views… is going to be, if not replaced, at least assisted by AI, which will start to provide the same comprehensive advice. And perhaps often it will be just an oncologist then, assisted by AI, that makes the decision, ultimately together with the patients, rather than having this [multidisciplinary] conference.*

[P4]

Though AI tools may be costly, the cost is expected to be offset by the benefits.

#### J2: Clinical Benefits

Participants note that the use of AI tools leads to better cancer detection, allowing clinicians to identify smaller cancers at an earlier stage. For example, one participant hares that when the AI tool they are implementing in breast cancer screening was evaluated,

*They found 29% more cancers compared to when there were 2 radiologists, and they found slightly smaller cancers and clinically significant cancers at an earlier stage.*

[P13]

Increased accuracy results in lowered patient recall. Furthermore, a participant highlights that the advanced 3D imaging technology used in breast screening can provide better detection of cancers in dense breast tissue. Using AI tools in segmentation tasks is reported by a participant as yielding better organ structures and missing fewer structures compared with manual delineation by physicians. Participants also observe that AI tools reduce inter-observer variation and perception errors. The greatest benefit, one says, is to the patients, accruing from early detection of disease and potential complications, as well as improved, timely treatment. A participant makes a similar assertion:

*So, it [AI tool] helps the patient. It lowers the error in the exams.*

[P10]

#### J3: Continuity

Some participants indicate a willingness to change their AI tools due to unmet needs, high costs, and unacceptable error rates. There is also the possibility that other tools are now available on the market that offer better features, better prices, or possibilities to run on on-site servers. A participant put it this way:

*We would love some extra things that are not there, we are considering doing a new comparison… and see if there are new vendors… who are doing a better job.*

[P6]

However, others have reached satisfaction with their existing AI tools and have no plans to look for alternatives. Even though some participants observed that performance has remained consistent since their tools were evaluated and validated, they see continual and long-term validation of the tools as necessary to ensure ongoing reliability and effectiveness. One participant also notes that the first version of nearly any technological tool in a complex system, such as health care, is often imperfect, and better versions tend to emerge over time.

### External Systems

This theme reflects the influence of the external systems, that is, the environment, on the STS, and how they can enable or constrain AI integration efforts at the local level.

#### E1: Regulatory Influence

Participants highlight challenges related to regulations. One notes the complexity of regulating AI in medicine, particularly that models are learning from data and evolving over time, making it difficult to claim reproducibility of results. Others pointed to the lack of clear guidelines that pose a challenge to AI use. One participant states:

*I think it’s a problem to have unclear guidelines and guidelines that are difficult to follow. It sets the stage for people to try to circumvent them or ignore them.*

[P9]

Even when guidelines exist, they are said to lack the detail required to give sufficient guidance. Participants perceive inconsistencies in how regulations are applied. For instance, while institutions are required to submit a notice to the Medical Products Agency to perform a prospective evaluation of an AI application, there is no equivalent regulation obligation when purchasing and deploying an AI application in clinical practice. This gap in regulation creates a loophole for the adoption of AI tools that have not undergone proper evaluation.

#### E2: Vendor Stability

Whether AI vendors will remain viable in the long term could pose a threat to the adoption and sustained use of AI tools. A participant expresses concern that some vendors may not have staying power. This is a risk for health care institutions that often seek long-term partnerships with technology providers. This uncertainty prompts the preference for in-house developed solutions.

*We can see, as a risk of course, for the moment. So many companies have popped up who provide us with solutions. Will they be here in 5 or 10 years, 20 years? You know… So, we need solutions that are reliable…*

[P1]

#### E3: AI and Clinical Guidelines

Participants reflect on how they perceive the influence of clinical guidelines and AI tools in cancer care. They acknowledge that AI could give recommendations that are not entirely aligned with existing guidelines, but could still offer better options for some patients: a flexibility that is considered acceptable given the mutable nature of clinical guidelines. Participants note that AI has the potential to help interpret the results of mega-trials and rapidly analyze the vast number of publications that inform guideline development. However, they also feel that AI tools remain inferior to the expert panels who create guidelines, and that AI tools should be optimized to follow guidelines while retaining their potential benefits. Participants also felt that clinical guidelines should acknowledge the increasing use of AI:

*I also think that the guidelines should mention it [the use of AI tools]… they need to acknowledge AI and give us some guidelines about when it’s feasible to use it or not, and some of the drawbacks that they have*.

[P15]

Participants also highlight the need for AI developers to stay up to date with guidelines to effect the necessary changes in model behavior, and it is seen as positive when vendors align their AI solutions with international guidelines and can tailor them to national guidelines.

#### E4: Macro-Level Enablers

As highlighted by participants, infrastructure and governance structures at the national level influence the implementation of AI tools. Technologically advanced countries that also have more centralized digital health systems, like Denmark, are viewed as better positioned to integrate AI tools in health care, as opposed to decentralized ones, like Sweden, that are perceived to have more challenges adopting AI tools. Participants point out the redundancy and cost associated with repeating the evaluation and validation of the same AI tool across different institutions and regions. They add that due to costs, not all regions use AI tools. They further suggest forming collaborations and cooperation between and within regions to promote mutual learning and optimize resources when exploring different AI solutions. One of them says:

*I hope that in Sweden we can establish a collaboration between different regions and between private and county council-funded care, where we collaborate on evaluations, because there are so many different solutions, and it is difficult to test all of them. It would be great if we could create some forum where we have common procedures on how to evaluate and how to share our experiences*.

[P2]

## Discussion

### Principal Findings

This study explores clinicians’ perspectives and opinions on the use of AI in clinical practice, with the aim of identifying key factors that shape clinician acceptance and AI integration into everyday practice. We sought to answer the questions: (1) How does the adoption and use of AI tools impact the clinical workflow in cancer care settings? (2) What factors influence clinician acceptance of AI tools in cancer care settings? To interpret the results, we draw on STS theory and UTAUT as complementary frameworks. We find that the successful integration of AI tools into clinical practice requires organizational support, careful consideration of potential effects on individuals, and alignment between technical offerings and the requirements of clinicians. While perceived ease of use (EE), PE, social influence, and facilitating conditions influence acceptance, attitudes, and trust are also expected to influence an individual’s BI and use of AI tools, suggesting an extension to the UTAUT model.

### Workflow Impacts and Sociotechnical Alignment

The introduction of AI tools in clinical practice changes the way that care is delivered. Understanding their adoption requires understanding their impact on clinical workflows. In this section, we use STS theory to understand how workflow dynamics change as AI tools are introduced. Participants note that the core goals for adopting AI are improving the quality of care and making efficient use of resources. To achieve these goals, there is a need to optimize clinical workflows while recognizing the interdependence between social and technical elements that comprise the work system.

From the social subsystem perspective, conditions positioning the organization for successful AI adoption include prior preparation, the uptake of robust AI tools and effective technical integration, increasing awareness, careful consideration of impacts on people and their daily practice, as well as feedback, adaptation, and continual validation. Introducing AI tools must take into consideration the local infrastructure, clinical practice, and institutional dynamics that shape and are shaped by their use. Organizational readiness provides a foundation for successful adoption and integration and requires both the capacity to deploy tools and to develop the necessary competencies [35]. Organizations must provide training, and, importantly, ensure that staff have adequate time for training [13]. The key to organizational readiness lies in aligning human roles, organizational processes, and technical functionalities to achieve both adaptation and value realization.

Local validation helps ensure that an AI tool performs adequately within a specific clinical context. It includes rigorous assessment to establish the AI model’s reliability, safety, and efficacy [36]. However, such validation efforts can be resource-intensive. This raises the need to reconsider how validation processes might be shared or collaboratively undertaken across institutions serving similar populations, thereby reducing redundancy and making the process more efficient. Validation has previously been discussed as a single event that occurs prior to the tool’s use in clinical practice [37]. This study also highlights the need for continual validation to ensure that systems remain reliable over time, which is especially important as AI models continue to learn from data and undergo adaptations. Local, recurring validation allows institutions to assess both the performance of the tools and the unintended consequences. Consequences such as workflow disruptions, cost inefficiencies, and bias may hinder long-term use [38]. These assessments ultimately inform institutional decisions regarding the continued use or abandonment of an AI tool. Beyond such high-level decisions, the integration of AI brings visible shifts in daily practice.

Integration of AI into routine clinical workflows presents a challenge to effective AI adoption in health care [6]. Clinical workflows change due to the introduction of AI tools, but integration also requires clinicians to adopt different routines, thus shaping the nature of work. Such changes require that stakeholders be adequately involved and informed early on to clearly understand the roles fulfilled by AI tools and the context-specific benefits to be derived from them. This early engagement increases stakeholder buy-in [37,39]. As our results reveal, there are different workflow impacts in different contexts. In breast cancer screening, apart from handling image analysis, the introduction of AI tools also alters the timing and sequence of clinician involvement, necessitating localized rethinking of roles and responsibilities, and prompting reallocation of expertise, such as that of junior and senior radiologists. Even when performing the same type of clinical task, institutions may adopt distinct workflows. The selection of an appropriate workflow is important, as variations in workflow design can lead to differences in clinical effectiveness and implementation success [40]. The speed of processing by AI tools produces results more quickly, which also alters expectations around when and how subsequent tasks are performed. Where segmentation of structures is concerned, AI tools perform the segmentation process that was previously handled manually by radiologists. Radiologists review the AI-generated structures and rectify those that are incorrectly produced. Thus, an asynchronous collaboration between radiologists and AI tools is formed. This collaboration results in more consistent structures and helps reduce inter-observer variability. It also maintains a closer alignment with clinical guidelines. However, participants also highlight that the impact of AI tools varies depending on imaging modality and clinical protocols used. For example, workflows differ depending on whether CT, MRI, or other imaging systems are used. For AI tools to be efficiently incorporated into health care practices, they must be designed with these practices in mind [7]. While many current AI users believe the workflow changes are not drastic, integration of AI tools can be challenging, resulting in workflow disruptions. Efforts should be made to integrate AI tools seamlessly into clinical workflows, as workflow disruption can increase cognitive effort and time for performing a task [41] and hinder adoption [8]. While several researchers highlight the importance of seamless integration into existing workflows, institutions may need to embrace structural changes and new clinical workflows [42].

In this study, we find that the majority of participants were not involved in the design of the AI tools acquired by their institutions, and that several users report that the tools did not fully address their needs. Tools developed without sufficient input from the clinicians who will use them tend to fail to deliver clinical value [43]. Better alignment can be achieved through early involvement of health care professionals in design activities, as useful AI tools ideally involve clinicians who can clearly define relevant use cases, based on their needs [7,43]. Nonetheless, feedback and adaptation, which involve health care professionals communicating with AI tool providers, help with aligning the AI tools with user needs. However, it is done retrospectively. This is important in STS theory, which emphasizes the significance of the interactions between people and technologies in the design and redesign of systems, key to the pursuit of optimization [44].

While the introduction of AI reshapes clinical routines and requires adjustment from clinicians, it also delivers tangible benefits that can improve workflow performance and clinical outcomes. Having put AI tools to use, several benefits are identified, such as time-saving, increased work efficiency, workload reduction, reduced inter-reader variation, increased tumor detection accuracy, and reduced patient recall. The benefits demonstrate that the integration of AI tools supports and enhances existing workflows, benefiting both clinicians and patients. The appreciation of AI tools despite some shortfalls attests to their utility. The realization of benefits, along with the participants’ view that great human-AI synergy arises when AI tools are integrated with experienced clinical judgment, aligns with STS’s understanding of co-adaptation between humans and technology.

AI tool design and integration should consider human factors [45,46]. The perceptions and opinions of health care professionals have given further insight into pressing concerns associated with AI adoption and use, such as trust, responsibility and accountability, interpretability and explainability, autonomy, and threats to jobs, skills, and competencies. We find that health care professionals are largely optimistic that the use of AI tools will yield positive returns for the health care industry overall. It must be noted that tensions exist between the perceived utility of AI tools and the commitment to maintain clinicians’ personal autonomy, where fear of displacement and the desire to preserve professionals’ authority have been highlighted. Autonomy extends beyond decision-making to encompass the preservation of clinical judgment, which is deeply informed by formal training, accumulated experience, and tacit knowledge acquired through practice. Verma et al [7] find that the clinicians’ need to maintain control over AI is raised in relation to responsibility and trust but is driven by their service to patients. Similarly, our results show that most health care professionals do not perceive accountability as a dilemma; they take full responsibility for patient assessments even when supported by AI tools. They emphasize that AI tools are used only as support and that physicians take the final decision regarding a patient and remain responsible and accountable to the patient. The threat of job loss also does not appear to be a significant concern to most in the short term. An understanding of how AI tools function and their current limitations provides health care professionals with assurance that widespread job displacement is not imminent but may be a concern for the future. To date, AI tools fulfill narrow-based tasks, whereas clinicians have expertise to handle diverse types of data modalities and have a holistic view of different diseases and patient situations [43]. Nonetheless, there is fear that fewer opportunities will exist for junior professionals and administrative workers. Although the threat may be distant for some, the concern about job security must be addressed [47]. Participants also foresee that skills may erode due to overreliance on AI. Increased reliance on AI tools means that they will have reduced hands-on practice, which may hinder their ability to perform certain tasks independently. Maintaining such skills is necessary if clinicians are to continue to oversee and verify AI-generated results. Measures, such as clinicians occasionally handling clinical tasks without AI assistance, are proposed. On the contrary, clinicians may have to adopt a different type of thinking, accepting that roles and responsibilities shift, and that they may no longer need to possess skills for certain tasks that AI tools execute exceptionally.

Trust in AI tools remains a complex issue. Although AI perceptions amongst clinicians appear largely positive, trust continues to be a concern for many. The novelty of AI in health care, lack of awareness, and uncertainty about AI’s long-term impact contribute to issues of trust. Closely linked is the perceived threat to clinician autonomy, confidence in their own work, and impact on interpersonal relationships. Lack of explainability is also linked to diminished trust [7]. Trust or lack thereof influences the adoption of AI tools and human-AI collaboration [47,48]. We also find that over time, however, skepticism appears to lessen with increased exposure, experience, quality control, and demonstrated performance of AI tools. While a lack of trust results in low adoption, trusting AI tools uncritically leads to overreliance and automation bias. Automation bias, along with other cognitive biases, can undermine the effectiveness of human-AI collaboration that is essential for optimal clinical outcomes [48]. This underscores the need for further research into the implications of too much reliance on AI tools and approaches to mitigate it. As participants noted, clinicians may occasionally accept AI-generated outputs without critically reviewing them, such as neglecting to verify the results independently or allowing the AI tool’s output to unduly influence their clinical judgment, undermining confidence in their expertise, and increasing patient risk.

Regarding the actual technology, participants view AI tools as having great potential to help improve health care delivery. Achieving this requires alignment between the design and performance of the AI tool and the individual or organizational goal to accomplish a specific task. Explainability and interpretability are well-known challenges in medical AI, touted as posing a significant barrier to the adoption of AI tools in clinical workflows [49]. Explainable AI offers transparency regarding how AI models reach their outputs, enables error analysis, and increases clinician trust [49]. Although interpretability and explainability are clearly desirable, in contexts such as image diagnostics, their lack does not appear to be a significant impediment since doctors can themselves perform the same tasks manually. This allows them to verify, accept, or refute AI-generated results. In line with this, Aung et al [9] argue that since clinicians use technologies such as CT and MRI, whose internal mechanisms they may not fully comprehend, they may also somewhat accept AI tools that lack transparency if their efficacy is proven. Nevertheless, AI tools can identify anomalies that are invisible or inexplicable to human experts, resulting in a lack of consensus between the clinicians and the AI tools. Lack of consensus is not new and can happen between clinicians. In the same way, there are protocols in place to address human-AI disparities. Still, AI as a technical subsystem presents a challenge to the transparency of tool operations, negatively impacting users’ understanding and trust in a tool’s given outcomes. However, the social subsystem, like in the case of physicians in image diagnostics areas, fills in this gap using their expertise, illustrating how STS relies on the interplay between human insight and technical function. It is worth noting that explainability remains a critical requirement for AI-based clinical decision support systems in oncology [50]. In fact, explainable and interpretable tools would be advantageous in many contexts, but they may not always hinder tool use.

Although data-related challenges are coded under the technical subsystem in our results, this discussion elaborates on how these issues are embedded within broader sociotechnical dynamics. One key data-related concern highlighted by participants is privacy and security, a concern also explored by Momani [51] and Gawankar et al [52]. While compliance with regulations such as the General Data Protection Regulation (EU) and Health Insurance Portability and Accountability Act (US) may address data privacy and security concerns, the other risks associated with the dependence of AI models on data are inherently sociotechnical in nature. Issues of data generation, availability, and correctness cannot easily be disentangled from the social system. Failed AI implementations also do not necessarily imply technical flaws, as inaccurate outputs may stem from an AI tool itself, poor-quality data input, or misalignment with clinical routines, contributing to suboptimal system performance. Another significant concern that arises from the AI models’ reliance on data is the propagation of bias, a phenomenon also documented in literature [53,54]. The issue of model bias warrants further scrutiny. Kotter and Ranschaert [43] argue that addressing this challenge requires standardized and regulated monitoring of outcomes.

Beyond the technical and social factors influencing AI adoption are broader external environmental factors. Participants expressed concerns about current regulatory frameworks for medical AI. They emphasize that regulations fail to account for the adaptive nature of AI algorithms, challenging the reproducibility of AI results. This is underscored by Chua et al [50], who also highlighted the need for a regulatory framework that acknowledges AI’s self-learning characteristic. The ambiguity and complexity of regulatory requirements, as well as inconsistent enforcement, create loopholes that may be detrimental to clinical practice. There is a need for clear guidelines that offer sufficient detail and are easy to interpret and implement in real-world health care settings. Additionally, the stability of AI vendors may significantly influence their sustained use. Participants express concern about the longevity of AI vendors, a concern that causes uncertainty since institutions show a preference for long-term partnerships with their suppliers. Continued use of AI tools requires adaptation and technical support, which depend on reliable vendor engagement. It is essential that vendors remain operational and maintain strong relationships with the institutions using their products. Failure to do so would lead to reduced user confidence and eventual tool abandonment. Although developing in-house solutions may be an alternative, health care institutions must be cognizant of the distinct challenges associated with such an approach [37,43].

Based on the shared perspectives, it can be inferred that the discussed social aspects are shaped by the views and performance of the technical system. Clinician behaviors evolve over time in response to using AI tools, while the tools themselves adapt through feedback to better meet the needs of clinicians, illustrating a mutual shaping of the social and technical systems. The main principle of the STS theory is that systems are most effective when the social and technical subsystems are jointly optimized to support a system’s objectives, such as improving the efficiency of clinical processes. To achieve this outcome, there must be a good fit between the subsystems, which facilitates seamless integration of AI tools into clinical practice. A well-optimized system requires a careful balance between tools that provide value while preserving enough clinician involvement to mitigate concerns of loss of control, which could hinder AI acceptance. Viewing AI through the lens of STS theory emphasizes the need to maintain the human-in-the-loop approach. This is particularly relevant given that our results suggest that participants perceive an optimal state of working when both an AI tool and a health care expert function together in a complementary manner.

### Acceptance of AI Tools by Clinicians

#### Overview

**Figure 1.**
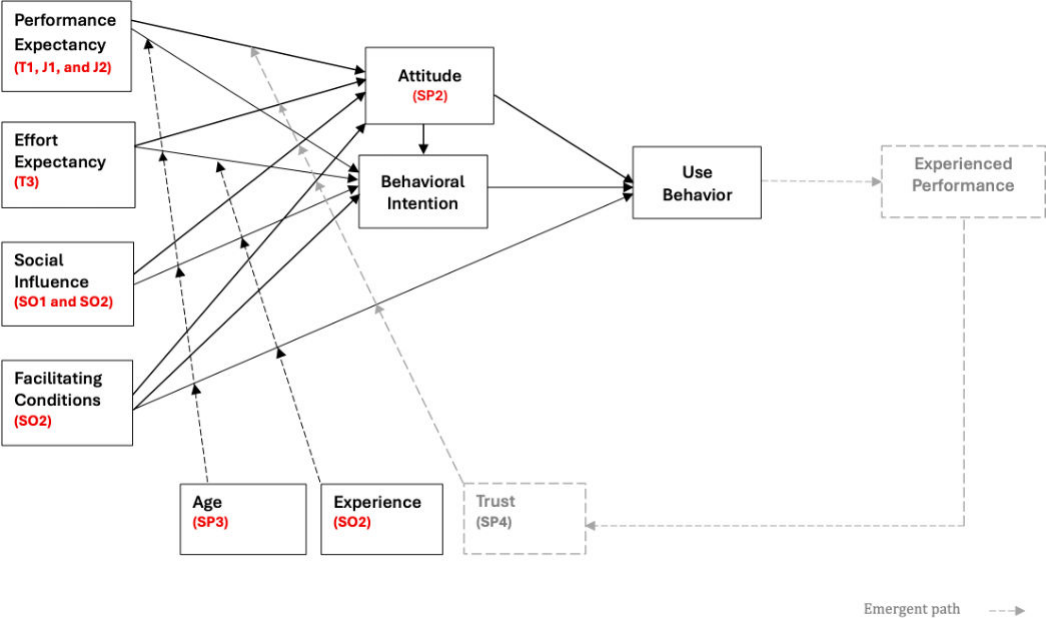
UTAUT diagram, with relevant subthemes between the parentheses in red (adapted from Dwivedi et al [55], which is published under Creative Commons Attribution 4.0 International License [56]). The moderators (age and experience) are as per the original UTAUT model by Venkatesh et al [27].

To answer the question of acceptance, we turn to the UTAUT model designed for understanding user acceptance. While themes aligned with the STS lens, some of our results reflect the constructs of UTAUT. UTAUT explains factors influencing BI, which in turn influence the use of technology [27]. The STS view of adoption, covering technical capabilities, user perceptions, and social factors, aligns well with the UTAUT constructs. Our results, on the other hand, also include actual use, as some participants have already begun using AI tools, and the use of technology is a strong signal of acceptance and successful integration. Figure 1 presents the UTAUT model, depicting the supporting factors according to our results, with relevant subthemes from the TA in parentheses, for example, T3 below.

#### Effort Expectancy (T3)

Our findings show that participants perceive AI tools to be easy to use. As a result, individuals are likely to accept them [14,27]. These findings are in line with several studies identified in a recent literature review by Madan et al [8]. Indeed, some views suggest that some level of difficulty exists. Still, these views are accompanied by expectations that such challenges only happen at the initial stages after the AI tools are introduced, but tend to diminish in the long run. Results also indicate that understanding the tools does not require intense training.

#### Performance Expectancy (T1, J1, and J2)

The results show that AI tools are perceived to be useful. Perceived usefulness [14], also known as PE [27], is a strong predictor of BI to use a technology [27,57]. PE can be drawn from our results covering the potential for AI tools and the benefits of their use. The results show that participants expect and even experience increased performance with AI tools integrated into clinical practice. AI tools have the potential to streamline care and alleviate pressure on strained resources, provide informational support for patients, and support knowledge development. Participants report that AI tools align well with the tasks they need to perform and contribute to increased accuracy, reduced workload, reduced inter-reader variation, reduced perception errors, time savings, and efficient work processes.

Additionally, using AI tools is seen as a safeguard, likely to increase their awareness of potential human errors. These tools support clinicians’ performance and benefit patients through early tumor detection, better treatment, and reduced recall. Given the overarching goal to provide high-quality care while optimizing resource use, these perceptions underscore the importance of EE in shaping positive attitudes and BI to use AI. These participants’ perceptions are critical as successful adoption of technologies often depends on how individuals perceive their relevance and utility [58]. While our results indicate BI to use AI tools, the realization of the outcomes mentioned above also confirms actual usage.

#### Social Influence (SO1 and SO2)

Our results on social influence highlight that information exchange occurs through informal professional interactions. These discussions are positive in nature as they revolve around best-performing tools for specific use cases, as well as the visible benefits derived from the AI tools that are introduced, which are likely to influence or even reinforce their intention to use them. Since many clinicians face time constraints and huge workloads, AI tool benefits such as time-saving and workload reduction can encourage their acceptance [13]. Social influence is expected to have a more substantial effect on BI where tool use is mandated rather than voluntary [27]. Apart from facilitating formal communications, AI champions can also be great influencers.

#### Facilitating Conditions (SO2)

Several facilitating conditions have also been mentioned by the participants, such as validation of tools, organizational communication, and integration with other technical infrastructure. Facilitating conditions extend to ensuring access to the necessary knowledge [59]. Availability of training and support facilitates the use of AI tools and has been highlighted by participants. Proper training on how to use AI tools increases confidence and encourages acceptance of AI tools [13]. However, we also find that, in some contexts, AI tools are seen as intuitive enough to integrate and use without prior training. This may be particularly true in technically inclined specialties where the introduction of AI tools can be easily integrated into existing routines. In other contexts, however, concerns are raised regarding how well tools integrate with other existing platforms, which deters from the willingness to continue using them.

#### Moderators (SP3, SO2, and SP4)

Age is expected to play a role in the willingness of health care professionals to accept and use AI tools. While this is not reported in first-person terms, discussions suggest that there is a difference between how younger professionals perceive AI compared with older professionals. Age has already been proven to have a moderating effect between the first 3 constructs and BI [27] regarding technology acceptance. However, its impact in relation to AI tools should be further investigated. Exposure and experience with AI tools, even prior to joining health care institutions, are expected to positively influence the willingness to use AI tools in clinical practice. Participants expect younger professionals to have had more exposure and experience with AI tools, both in educational and personal settings. Apart from this, our study suggests that clinicians who work in tech-heavy specialties, while critical towards how AI tools perform, display less resistance towards AI use. Future research can elucidate whether the experience of technology use in general plays a role in individuals’ BI to use AI tools.

#### Attitude (SP2)

Attitude, which had been included initially as a construct in the TAM, was not included in UTAUT after being found to be redundant as a predictor of BI. Dwivedi et al [55] reintroduced attitude to their UTAUT adaptation, finding that attitude plays an important role in acceptance and use of technology. In turn, attitude is influenced by EE, PE, social influence, and facilitating conditions. The latter is especially true in nonvoluntary settings. Our results reflect a more positive attitude than negative ones, as well as a keenness to use AI tools. According to Dwivedi et al [55], users with a positive attitude are more likely to use the technology; thus, attitude influences both BI and usage behavior. As earlier established, trust (SP4), or lack thereof, can impact whether individuals accept technology. The issue of trust is more pronounced in the context of AI tools, where functionality demands more autonomy and offers less transparency. Trust is likely to have a moderating effect between PE, EE, SI, and FC and an individual’s attitude towards AI tools. We have also learned that experienced positive performance reduces skepticism and increases an individual’s level of trust. Further research can elucidate the hypothesized effect of trust. A recent study [60] finds that age and experience do not significantly influence attitude.

The alignment of the data, as shared by participants, with UTAUT constructs suggests a strong foundation of acceptance of AI tools in cancer care, while also highlighting potential hindrances. The already established use of AI tools, accompanied by a broadly positive attitude, reflects acceptance and supports UTAUT’s outcome of use behavior. However, continued use is contingent on consistent performance of AI tools, adaptability, and addressing unmet needs. While UTAUT helps us understand individual acceptance, its constructs clearly show the interplay between the social and the technical aspects of an STS.

PE, while seemingly technical, is not only about AI’s capabilities and its usefulness, but it is also shaped by social perceptions and experiences. Performance of AI tools shapes how individuals perceive them, and perceptions of usefulness, in turn, influence the social aspect, human BI. EE may also appear to be a social aspect since it pertains to how people feel about using the technology and the effort required from them; however, it is influenced by the tool design, interface, and technical support. When AI tools are effectively integrated into clinical workflows, they are more likely to reinforce the perceptions of their usefulness. Social influence is more obviously tied to the social subsystem: people, relationships between them, and social norms. Facilitating conditions entwine the social and technical systems in that they encompass training and other factors relating to organizational preparedness, as well as technical infrastructure, interoperability, technical capability, and fit. These constructs influence sociotechnical outcomes: BI and use. This overlay between UTAUT and STS signifies how AI acceptance is shaped by the interplay between AI tools and the social system that they are intended to support. From an STS perspective, the relationship between user acceptance and the successful integration of AI tools in clinical workflows is bidirectional. Well-integrated AI tools that align with clinical tasks, provide utility, and minimize disruption can foster clinician acceptance [13,50]. However, clinician acceptance can also facilitate a smooth integration of AI tools into the clinical workflow. Openness and willingness to engage with AI tools enable learning, engagement in feedback and adaptation, and meaningful use.

### Limitations

Recruiting participants with the specific profile required in this study was challenging due to their limited availability and demanding schedules. Using purposive sampling, we attempted to recruit 135 potential participants. Although efforts were made to recruit participants from a range of medical specialties within the set criteria, and without emphasis on proficiency levels, there remains a possibility of selection bias, as researchers cannot guarantee reaching all relevant individuals. Additionally, the voluntary nature of participation may have also introduced self-selection bias, whereby those with a stronger interest in AI were more likely to take part. We do not claim generalizability of these results to all oncology clinicians. However, the participants’ time constraints and scarcity in research participation make the contribution of the participants especially valuable, lending it a unique strength.

Our sample included 18 health care professionals involved in cancer care, encompassing both practicing clinicians and nonclinical roles such as biomedical technicians and medical physicists, as well as one participant from AI research and development, to capture a broader range of insights related to AI adoption and use in cancer care. Although not all participants were patient-facing clinicians, those in nonclinical roles play a critical role in implementing and managing AI tools, particularly in diagnostic imaging, radiation therapy, and treatment planning.

While the sample size is adequate for qualitative analysis, it remains relatively small and is drawn from specific health care institutions, primarily located in the Nordic region. This may limit the transferability of the findings to other contexts or countries. The majority of participants are based in Sweden, a technology-forward country. Such a positive view may bias the results towards more positive perceptions of AI tools. Additionally, the study focused on contexts where image-based AI tools are used. For this reason, issues that may be unique to cancer care contexts that use different data modalities could have been missed. However, this is not expected to invalidate the results, as literature shows that the most approved AI tools in cancer care are AI-based image diagnostic tools.

We acknowledge that the term “AI tool” may be perceived as too general. However, we maintain its use as an umbrella term because participants describe a variety of AI tools and even similar tools used differently within similar clinical domains. This diversity reflects real-world variation in AI implementation and related experiences across health care settings. As with all qualitative studies, the findings are intended to offer rich, contextual insights into the phenomena of AI acceptance and adoption and not statistical generalizability. Despite these limitations, this study offers a valuable perspective into the real-world application of AI tools in cancer care from a sociotechnical viewpoint.

### Conclusions, Recommendations, and Future Research Directions

Participants (primarily users/evaluators in settings where AI tools are present) generally perceive AI tools to deliver tangible benefits for both clinicians and patients. However, successful implementation requires careful integration into workflows, alignment with clinician needs, and continued validation. Applying STS theory, we offer a holistic view of how AI tools shape and are shaped by health institutions, people, and their work processes. Understanding this sociotechnical interplay is critical for AI tools that support clinicians in delivering quality care while preserving clinical expertise. Using UTAUT, we identify the determinants influencing the clinicians’ intention to adopt or continue using AI tools. Our findings suggest that acceptance is not only a precursor to use but can also result from how well AI tools integrate into and support established work routines and clinical needs. Although a general positivity towards using AI tools is seen, we highlight the need for health care institutions to pay attention to the concerns that negatively impact clinicians’ acceptance of AI tools, such as the need for communication early on in the project, performing an impact analysis to ascertain tool impact on people and workflows early on, increasing knowledge of AI and potential negative consequences, and carrying out continual validation.

More importantly, the newly arising negative effects of AI use, such as automation bias and potential skill erosion, must be addressed. Mitigation strategies, such as periodic non-AI proficiency runs where work should periodically be done independently and without the support of AI tools, have been suggested by participants to preserve skills. Another way is AI implementation that enforces double reading for edge cases to ensure that 2 clinicians attend complex or ambiguous cases and continue to apply and sharpen their skills.

External factors such as regulations, vendor stability, and broader infrastructure and governance at the national level can also facilitate or hinder adoption and sustained use. Current approaches to validation are perceived as onerous. The cost, time, data, and data preparation tasks, and effort to perform local validation for every trial AI product are not sustainable and delay AI adoption. As suggested by participants, shared regional validation consortia may be a solution. This may be a reasonable approach for regions within a single country or group of countries where a similar patient population is served. Within such a consortium, validation strategies, resources, and experiences can be shared, which may reduce costs and uncertainty and result in more robust, long-term implementations.

Future research should address training needs and how to implement safeguards against unintended consequences, such as overreliance and skill erosion, as well as prioritize co-design with clinicians to ensure better tool-task alignment.

## Supplementary material

10.2196/83240 Multimedia Appendix 1 Interview Guide.

10.2196/83240 Multimedia Appendix 2 Table S1 – Participant Characteristics.

10.2196/83240 Multimedia Appendix 3 Table S2 – Mapping of subthemes to STS theory components.

10.2196/83240 Multimedia Appendix 4 Codebook.

10.2196/83240 Multimedia Appendix 5 Consent Form.

10.2196/83240 Multimedia Appendix 6 Table S3 – Themes, sub-themes, and associated codes.

## Abbreviations

AI: artificial intelligence
BI: behavioral intention
CT: computed tomography
EE: effort expectancy
EHR: electronic health record
FC: Facilitating Conditions
MRI: magnetic resonance imaging
PE: performance expectancy
SI: Social Influence
STS: sociotechnical systems
TA: thematic analysis
TAM: technology acceptance model
UTAUT: Unified Theory of Acceptance and Use of Technology
