# Supplementary material for: Perceptions of Health Care Professionals on the Integration and Use of AI in Clinical Cancer Care: Interview Study
Source: JMIR Hum Factors. 2026 Apr 20;13:e83240. doi: 10.2196/83240 (PMC13094801; doi:10.2196/83240)
Supplement: Multimedia Appendix 1 [file humanfactors-v13-e83240-s001.pdf]

Starting the interview
Thank you for making time to meet us for this interview.
Introduce ourselves, research focus, affiliation to Karlstad University

Introduce the research study's purpose, why they have been approached, the interview process, recording, check if there are questions, and get consent (if not already submitted).

Interview Questions

1. **General**:
    a. Occupation - Can you briefly describe your work role?
    b. Do you use any AI tools in clinical practice? Or are you aware of any AI tools that are being used/evaluated in your institution (your and other cancer care related departments)?

    If yes continue, if not (see section 5 below):

    c. What AI-based tools do you use in your workplace? How often?
    d. What is the purpose of using X? (may need to ask Y? Z? depending on the no. of tools listed above)
    e. How long has it been in use in clinical practice?
    f. How did you get to know about the institutions intention to acquire X?
    g. Was any relevant training given regarding X? Having used X, have you had thoughts about ways training could have been improved to better suit your needs?
    h. To what extent does the information shared / training cover potential negative consequences of using AI?
    i. As a (occupation) to what extent were you involved in making the decisions regarding the adoption of X?

2. **Involvement in Evaluation-Validation**

Many AI tools undergo an evaluation-validation period before being used in clinical practice.

    a. To what extent were you involved in the evaluation-validation of the AI tool?

    (If involved)

        i. How has involvement in the evaluation-validation impacted how you perceive the use of X in daily practice?
        ii. Do you use X in the same way in daily practice as you did at evaluation?
        iii. Has the performance of X remained the same now that it is part of daily use in clinical practice?
        iv. Have any issues arisen during use in clinical practice that were missed during evaluation-validation?

    (If not involved)

        v. Are you aware of the evaluation-validation that was conducted?
        vi. How were the results of such an evaluation shared?

3. **Experiences using X**
    a. How has the introduction of X impacted your way of work?
        i. Has the use of X necessitated any changes in the way you work?
            - Has the use of X necessitated any changes in the way you capture/enter patient data for example
        ii. How do you feel about the 'changes' (if mentioned)
    b. Do you feel it is beneficial/useful to use X?

i. Can you share any benefits/useful ways that you (and the healthcare institution) have realised or expect from using X?
ii. If not, can you elaborate why?

c. Has your workload changed following the introduction of X, if so how? If not, why?
d. Do you interact with the tool directly or just its output (e.g. the result that has already been generated by an AI tool)?
   i. Tool: Do you find it easy to understand and use?
   ii. Tool: Do you find it easy to recover if mistakes or errors happen?
   iii. Output: How effective/useful is it?
e. Is there any kind of support in place to turn to when help is needed?
f. Is it possible for you to see what features the AI model used to reach its decision (prediction) in contrast to a black-box model?
g. How necessary is it for you to understand how the AI tool has reached its decision (transparency)?
h. Have you experienced any challenges using X? How are they overcome?
i. How would you say your overall experience working with AI has been?

4. Round up

a. Do you have any concerns about X? If yes, please elaborate.
b. What do you believe will be the impact of AI in general in the healthcare system?
c. How do you think the skills of healthcare workers will be the impacted by the use of AI in healthcare?
d. In your view, would new and changing clinical guidelines affect how AI is used in healthcare? In what ways?
e. Can you think of examples of ways in which discussions amongst colleagues about AI influence its acceptance?
f. Can you share insights or recommendations that can help to ensure smooth integration of AI into healthcare?
g. Is there anything else you would like to share?

---

5. No AI use, and not currently considering/evaluating one

If no (to Q1b above)

a. Are you aware of how AI tools currently used in cancer care (according to specific area of care within the medical field)? Can you tell us a bit about that?
b. Has your institution/department considered using AI tools, if so, can you expand on your experiences?
c. Generally, what benefits, if any, do you believe can be derived from using AI tools in your area of work?
d. Generally, what concerns, if any, do you have about the use of AI tools for cancer care?
e. AI tools are often evaluated at the healthcare institution before they are used in clinical practice. In what ways do you believe that involvement of clinicians in such evaluations (as opposed to only training) would impact clinicians' use of the tools in practice?
f. How about involvement in design? would it be possible for them to have a say?

g. Based on your knowledge of clinical work and understanding of AI use, how do you think the way of work would be impacted by the introduction of an AI tool?
h. In your view, how important is it that healthcare professionals understand how the AI tool has reached its decision?
i. What do you believe will be the impact of AI in general in the healthcare system?
j. What impact do you believe AI would have on the skills of healthcare professionals in this area?
k. What are your views on informing and giving patients the opportunity to consent to AI use when AI is used in clinical decision-making?
l. How do you foresee the interplay between following clinical cancer care guidelines and AI recommendations (the potential for differences between them)?
m. What other ways can AI tools can be used in cancer care?
n. Is there anything more you'd like to add?
