# Supplementary material for: Perceptions of Health Care Professionals on the Integration and Use of AI in Clinical Cancer Care: Interview Study
Source: JMIR Hum Factors. 2026 Apr 20;13:e83240. doi: 10.2196/83240 (PMC13094801; doi:10.2196/83240)
Supplement: Multimedia Appendix 2 [file humanfactors-v13-e83240-s002.docx]

*Table A1: Participant characteristics*

| **Characteristics** | | **No. of participants** |
| --- | --- | --- |
| **Field of work** | Medical | 18 |
|  | Medical AI research & development | 1 |
| **Specialty**^a^ | Radiology | 9 |
|  | Oncology | 5 |
|  | Dermatology | 2 |
|  | Surgery | 1 |
|  | General Medicine | 1 |
|  | Immunology, genetics & pathology | 1 |
|  | Research & Development | 1 |
| **Location** | Denmark | 2 |
|  | Finland | 1 |
|  | Brazil | 1 |
|  | Sweden | 15 |
| **Gender** | Female | 9 |
|  | Male | 10 |
| **Level of AI exposure**^b^ | Using AI tools in clinical practice | 11 |
|  | Evaluated/Evaluating AI tools for use in clinical practice | 11 |
|  | Neither engaged in evaluation or use of an AI tool | 4 |

^a: There were 19 participants. One participant is employed in both Radiology and Oncology.^

^b: Level of exposure is not mutually exclusive; some current users were/are involved in evaluating tools and others not.^

^Similarly, some are/have been involved in evaluation but not use, while others are involved in neither.^
