# Supplementary material for: Perceptions of Health Care Professionals on the Integration and Use of AI in Clinical Cancer Care: Interview Study
Source: JMIR Hum Factors. 2026 Apr 20;13:e83240. doi: 10.2196/83240 (PMC13094801; doi:10.2196/83240)
Supplement: Multimedia Appendix 3 [file humanfactors-v13-e83240-s003.docx]

*Table A2: Mapping of sub-themes to STS theory components*

| **STS component** | **Description of STS component** | **Relevant sub-themes** | **Description of sub-theme** |
| --- | --- | --- | --- |
| **Social Subsystem (Organization)** | *The social subsystem* refers to the social elements present in a sociotechnical system. Within an organization, this includes the structures and people in that organization.  Separating the *organization* aspect allows us to capture the organization-specific factors that may influence the adoption of AI tools | Organization-specific challenges to adopting AI | This sub-theme captures the organizational level challenges put forward by participants, that can impact adoption and user-level acceptance of AI tools |
|  |  | Facilitating factors at the organization level | This sub-theme is concerned with those factors at organizational level that may enable AI tool adoption and use |
|  | Several factors at the organizational level, such as communication (or lack thereof), procurement challenges, bureaucratic processes, conducting the necessary validation etc. pertain to information about the organizational structure and processes the organizations follow (social subsystem) that may hinder or enable the adoption and use of AI tools and impact the overall sociotechnical system. This subsystem is responsible for allocating the resources and driving change – factors that play key roles in the sociotechnical system. | | |
| **Social Subsystem (People)** | The *social subsystem* includes the people within an organization, relationships between them, and other attributes and work-related elements  The *people* aspect allows us to capture human-specific factors that may influence the acceptance and use of AI tools | Need for AI awareness | Captures the identified need for awareness, knowledge, and training |
|  |  | Attitudes towards AI | Refers to the clinicians’ disposition towards AI tools |
|  |  | Age-related perceptions | Reflects perceptions on how age appears to influence disposition towards AI tools |
|  |  | Trust | Factors that influence clinician trust in AI tools |
|  |  | Involvement in shaping the solution | Perceptions on involvement in the design and continued adaptation of AI tools once implemented |
|  |  | Responsibility and Accountability | Shared thoughts regarding to whom responsibility and accountability falls for the AI-supported decisions and their potential impacts |
|  |  | Automation bias | Perceived impacts of AI tools on clinicians’ actions and decision-making |
|  |  | Clinician autonomy, behavioral impacts, and interpersonal factors | Reflects the perceptions about the impact of AI tools on clinicians’ sense of control, working relationships with others, as well as how other fears may influence behaviors. |
|  |  | Effect on jobs, skills, and competencies | How participants perceive that AI tools will impact jobs, skills and competencies |
|  | Within the social subsystem are people, whose level of awareness, attitudes towards AI, age, trust, needs-tool alignment and concerns regarding responsibility, and accountability, as well as autonomy may lead to lower acceptance and use. Human-related factors play a key role in the sociotechnical system since without people, the social system does not exist. Negative views regarding these factors can significantly impact the success of adoption efforts | | |
| **Technical Subsystem** | *The technical subsystem* refers to the tools, techniques, and processes that the workers need to fulfil organizational tasks and objectives  The specified categories pertain to participants perceptions of technology | AI potential | Expectations of participants of AI tools |
|  |  | Fit-for-task design | Participants’ views on the suitability of AI tools to the tasks they need to perform |
|  |  | Ease of use | The extent to which participants find the AI tools easy to use |
|  |  | Challenges related to data | AI tools are dependent on patient data. Access and use of these data may raise data privacy and security concerns |
|  |  | Interpretability and Explainability of AI tools | Interpretability and explainability of AI tools aid understanding of how AI tools reach decisions which better support clinicians’ decision-making processes. This sub-theme captures participants views regarding AI tools’ interpretability and explainability |
|  |  | Unsuccessful AI efforts | Participants’ experiences with AI tools that have failed on implementation |
|  |  | Effects of AI tools on workflow | Covers how AI tools complement or support clinicians’ tasks and activities within their workflows |
|  | The sub-themes that form the theme technical subsystem discuss how AI tools are perceived by participants. The sociotechnical system requires that both the technical and social systems work well together. The technical factors discussed in these sub-themes show what people (the social system) expect from AI tools, the challenges that arise, and the impacts of their use. People reflect on technical factors in relation to themselves (e.g., how easy-to-use they perceive them to be) and how they impact their workflow, as well as the features that may impact their trust and specific use patterns e.g., interpretability and explainability of AI tools. | | |
| **Joint Optimization (Impacts of AI Integration as Indicators of Joint Optimization)** | A key principle of STS theory is that both the social and technical systems must work together in a complementary manner to achieve a mutual goal. | Benefits to clinical practice | Captures perceived benefits related to actual work processes |
|  |  | Clinical benefits | Captures perceived benefits related to patients |
|  |  | Continuity | Refers to those actions that result in socio-technical alignment and continued use (or not) of AI tools |
|  | A jointly optimized system should result in attaining the main goals for introducing new technology in the work system, e.g., reduced workload (clinical practice) and accuracy detecting cancers (patients). To ensure continuity, the AI tools must continue to serve the work system. If the technical subsystem does not perform well enough to support the clinicians, or clinicians use of AI tools effectively, the system is not jointly optimized. These three sub-themes are indicators of joint optimization; without which they would be negatively affected. | | |
| **External Systems** | External systems refer to the environmental factors outside of the organization that influence the work system | Al and clinical guidelines | Clinical guidelines offer recommendations for care treatment. The sub-theme covers whether and how participants believe AI tool use will impact or be impacted by clinical guidelines |
|  |  | Macro-level enablers | This sub-theme covers those governance level factors that may impact either positively or negatively the diffusion of AI tools amongst institutions, regions, and countries |
|  |  | Vendor stability | Refers to the ability of the companies that supply AI tools to continue to thrive in the market |
|  |  | Regulatory influence | Regulations relating the AI development and its use, including access to data for this purpose |
|  | External factors such as clinical guidelines, vendor stability, regulations and other issues at the macro level impact the adoption and continued use of AI tools, and thus it impacts the sociotechnical system. When guidelines and regulations change, they can impact how AI tools are used. Stable vendors can provide continued support and updates for AI tools, increasing trust, and their continued use. | | |
