# Supplementary material for: Perceptions of Health Care Professionals on the Integration and Use of AI in Clinical Cancer Care: Interview Study
Source: JMIR Hum Factors. 2026 Apr 20;13:e83240. doi: 10.2196/83240 (PMC13094801; doi:10.2196/83240)
Supplement: Multimedia Appendix 4 [file humanfactors-v13-e83240-s004.pdf]

# Study on Clinician Acceptance & AI Integration – Codebook exported from NVivo

## Codes

| Theme/Description | *Subtheme*<br>Code Label | Sources | References |
|---|---|---|---|
| **1. General AI adoption perceptions** (General perceptions of AI and related developments in relation to health- and cancer care) | | 13 | 31 |
| | ***AI applications (for reference-intro)*** | 9 | 16 |
| | AI for communication with patient | 1 | 1 |
| | AI for consolidating data | 1 | 1 |
| | AI in pathology for biopsy and genetics exams | 1 | 1 |
| | AI tool for prostate cancer | 1 | 1 |
| | AI tool use in surgery for suturing | 1 | 1 |
| | AI-based image diagnosis tools | 2 | 2 |
| | areas for which AI tools can help | 1 | 1 |
| | LLMs for analyzing gene sets | 1 | 1 |
| | pancreatic cancer scanners with AI possibly | 1 | 1 |
| | other relevant AI applications | 1 | 3 |
| | solutions under consideration | 1 | 1 |
| | speech recognition for taking journal notes | 2 | 2 |
| | ***AI developments*** | 8 | 10 |
| | developments have been slower than initially expected | 3 | 3 |
| | expects upgrades will result in less mistakes | 1 | 1 |
| | fast moving field | 1 | 1 |
| | healthcare should take a more proactive focus | 1 | 2 |
| | part of societal development | 1 | 1 |
| | surprised at the limited use | 1 | 1 |
| | upcoming uses including LLMs | 1 | 1 |
| | ***Limitations and complexity*** | 3 | 5 |
| | complexity of AI in healthcare | 2 | 2 |

| | | | |
|---|---|---|---|
| | concern about future impact of AI | 1 | 1 |
| | uncertainty about the so-called-promise of AI | 1 | 1 |
| | what competition promotes is not always correct | 1 | 1 |
| **2. Social system – organization** (Perceptions about organizational level factors influencing AI adoption) | | 10 | 28 |
| | ***Facilitating factors at the organization level*** | 9 | 14 |
| | communicating with teams | 3 | 3 |
| | informal social discussions on AI | 2 | 2 |
| | prep work before implementation | 4 | 4 |
| | tech-savvy group, probably more willingness | 4 | 5 |
| | ***Organization-specific challenges to adoption*** | 5 | 14 |
| | challenges related to infrastructure and upgrades | 1 | 1 |
| | cumbersome process | 1 | 4 |
| | organizational guidance | 1 | 1 |
| | user buy-in | 4 | 8 |
| **3. Social system – people** **(**Perceptions about human and social factors that influence AI acceptance and use, as well as the broader impact of AI use on clinical practice and the workforce) | | 18 | 140 |
| | ***Age-related perceptions*** | 3 | 3 |
| | acceptance not correlated to age | 1 | 1 |
| | age specific difference in attitude | 1 | 1 |
| | younger professionals expected to know more | 1 | 1 |
| | ***Attitudes towards AI*** | 12 | 22 |
| | Negative attitudes towards AI | 4 | 4 |
| | Indifference | 1 | 1 |
| | negative because they felt were not informed | 1 | 1 |
| | skepticism overcome by AI performance | 2 | 2 |
| | Positive attitudes towards AI | 9 | 18 |
| | embracing AI as a helper | 1 | 1 |
| | generally satisfied | 5 | 8 |
| | less negative feedback than expected | 1 | 1 |
| | positive attitude also increased by AI for personal use | 2 | 2 |
| | positive attitudes driven by perceived benefit | 3 | 4 |
| | positive with a critical eye | 1 | 1 |
| | radiologists generally more positive | 1 | 1 |
| | ***Automation bias*** | 4 | 5 |
| | reviewer bias alignment with AI tool results | 2 | 2 |
| | physicians must check before approving | 1 | 2 |

| | | | |
|---|---|---|---|
| | younger drs believe AI results more, less scrutiny | 1 | 1 |
| | ***Clinician autonomy, behavioral impact, interpersonal factors*** | 7 | 13 |
| | concern that doctors lose their help from admin | 1 | 1 |
| | Dependency after continuous use | 3 | 4 |
| | fears associated with AI use | 3 | 5 |
| | human bias and error when AI is assumed to be wrong | 1 | 1 |
| | loss of control | 2 | 2 |
| | ***Effect on jobs, skills, and competencies*** | 15 | 41 |
| | Effect on jobs | 7 | 14 |
| | AI can take over mediocre jobs and tasks | 1 | 1 |
| | fear of job loss – admin | 1 | 1 |
| | great when supervised | 1 | 1 |
| | human involvement remains necessary | 5 | 7 |
| | impact on jobs will be evident several years from now | 2 | 4 |
| | Skills and competencies | 14 | 27 |
| | changes in medical training | 3 | 3 |
| | example of how skills weaken | 1 | 1 |
| | impact on younger generation of clinicians | 2 | 2 |
| | shift in responsibilities, need to reapply minds | 3 | 5 |
| | solutions for maintaining skills | 3 | 4 |
| | threat to epistemic knowledge | 1 | 1 |
| | training prior to AI tool use | 10 | 11 |
| | ***Involvement in shaping the solution*** | 12 | 23 |
| | desired design changes | 2 | 2 |
| | evaluation protocol incl drs opinions | 1 | 1 |
| | feedback and adaptation | 4 | 7 |
| | HCPs should be involved in design | 4 | 4 |
| | involvement during development | 3 | 4 |
| | no involvement in design | 1 | 2 |
| | no need to involve HCP in design | 1 | 1 |
| | Possibility of involvement in design is unclear | 1 | 1 |
| | user involvement through demonstrations | 1 | 1 |
| | ***Need for AI Awareness*** | 9 | 12 |
| | lack of awareness | 2 | 2 |

| | | | |
|---|---|---|---|
| | Misconceptions | 3 | 5 |
| | need to be alert to potential AI errors | 3 | 4 |
| | need to understand AI tools to use them correctly | 1 | 1 |
| | ***Responsibility-Accountability*** | 9 | 11 |
| | dr is always responsible | 6 | 7 |
| | question of responsibility | 3 | 3 |
| | responsibility of developer to make sure it works | 1 | 1 |
| | ***Trust*** | 7 | 10 |
| | issue of trustworthiness | 2 | 3 |
| | reliance more on experience than AI | 1 | 1 |
| | successful evaluation linked to increased trust | 1 | 1 |
| | trust based on certification and approval | 1 | 1 |
| | trust based on exposure and experience | 1 | 2 |
| | why should AI be treated differently to other tech | 1 | 1 |
| | would rather trust well-respected clinicians | 1 | 1 |
| **4. Technical (AI) system** (Perceptions of AI tools) | | 18 | 90 |
| | ***AI potential*** | 10 | 23 |
| | AI can be helpful for patients | 2 | 3 |
| | AI can help clinicians stay up to date | 1 | 1 |
| | can speed up research to implementation | 1 | 1 |
| | functional limitations | 2 | 2 |
| | streamline care, relieve strained resources | 8 | 16 |
| | ***Challenges relating to data*** | 7 | 8 |
| | concern for data privacy & security | 5 | 6 |
| | concern for propagating bias | 1 | 1 |
| | concern if humans capture data correctly | 1 | 1 |
| | ***Ease of use*** | 7 | 14 |
| | availability of support | 5 | 6 |
| | easy to use | 4 | 5 |
| | error recovery | 1 | 1 |
| | little slow at first, bit more work | 2 | 2 |
| | ***Effects of AI tools on workflows*** | 9 | 20 |
| | AI adaptation to suit workflow needs | 1 | 1 |
| | aids for workflow integration | 1 | 1 |
| | change in workflow | 7 | 14 |

| | | | |
|---|---|---|---|
| | Different types of AI solutions have different impact on workflow | 1 | 1 |
| | Modes of AI integration | 3 | 3 |
| | ***Fit-for-task design*** | 5 | 5 |
| | be specific to a given use case | 3 | 3 |
| | main goals warranting AI use | 2 | 2 |
| | ***Interpretability and explainability of AI tools*** | 9 | 13 |
| | interpretability benefits clinicians | 1 | 3 |
| | Lack of interpretability causes uncertainty and future concern | 1 | 1 |
| | need for explainability-interpretability | 2 | 2 |
| | need for interpretability is conditional | 2 | 2 |
| | tool is not interpretable, but not needed | 4 | 4 |
| | trade-off between accuracy and explainable | 1 | 1 |
| | ***Unsuccessful AI efforts*** | 3 | 7 |
| | custom-made systems offer minimal gains | 1 | 2 |
| | failed AI example (commercial product) | 1 | 1 |
| | failed AI example (in-house) | 1 | 1 |
| | Model robustness | 1 | 3 |
| **5. Impacts of AI integration as indicators of joint optimization (**Reflects the perceived outcomes following the integration into clinical workflows**)** | | 15 | 70 |
| | ***Benefits to clinical practice*** | 14 | 40 |
| | increased accuracy and reliability | 4 | 4 |
| | increases confidence in drs | 1 | 1 |
| | increasing efficiency, effectiveness | 6 | 12 |
| | potential to reduce the need for MDTs | 1 | 1 |
| | saved money (in terms of salaries) | 1 | 1 |
| | workload reduction and time saving | 10 | 20 |
| | ***Clinical benefits*** | 8 | 16 |
| | better cancer detection | 2 | 3 |
| | better structures | 1 | 1 |
| | patient benefits from better treatment | 3 | 4 |
| | reduce perception errors | 2 | 2 |
| | reduced variation | 3 | 3 |
| | ***Continuity*** | 8 | 14 |
| | consistent performance | 2 | 2 |

| | | | |
|---|---|---|---|
| | continuous validation | 3 | 3 |
| | decision to change solution or not | 4 | 4 |
| | drivers for considering change | 2 | 4 |
| | expectation that solution will evolve rather than discontinued | 1 | 1 |
| **6. External System - Environmental factors** **(**Addresses perceptions about the environment and its potential influence on AI adoption and integration efforts**)** | | 13 | 19 |
| | ***AI and clinical guidelines*** | 7 | 9 |
| | AI can help with analyzing mega trials for guideline development | 1 | 1 |
| | AI should be optimized to follow guidelines without compromising AI benefits | 1 | 1 |
| | AI today is much inferior compared to expert committees regarding clinical guidelines | 2 | 2 |
| | guidelines should acknowledge AI use | 2 | 2 |
| | manufacturers' alignment with guidelines | 2 | 3 |
| | ***Macro-level enablers*** | 4 | 4 |
| | centralized healthcare systems make AI implementation easier | 1 | 1 |
| | cooperation between regions | 3 | 3 |
| | ***Manufacturer stability*** | 1 | 1 |
| | ***Regulatory influence*** | 4 | 5 |
| | a challenge to regulate AI in medicine | 1 | 1 |
| | AI developments constrained by regulations | 1 | 1 |
| | legal barriers to AI use in clinical practice | 1 | 1 |
| | unclear guidelines are problematic | 1 | 2 |

*Note that the aggregated source count for each theme reflects unique sources. As sources can contribute to multiple codes, the sum of the code-specific counts can exceed the theme's source total
