# Supplementary material for: Perceptions of Health Care Professionals on the Integration and Use of AI in Clinical Cancer Care: Interview Study
Source: JMIR Hum Factors. 2026 Apr 20;13:e83240. doi: 10.2196/83240 (PMC13094801; doi:10.2196/83240)
Supplement: Multimedia Appendix 5 [file humanfactors-v13-e83240-s005.pdf]

Consent for participation in the project

I have received spoken and written information about the project and have had the opportunity to ask questions. I can keep the written information. I am aware that the collected data will be processed in the manner described in the research participant information sheet and

☐ I **consent** to participating in the project **Stakeholders' Perceptions on the Adoption and Use of AI in Oncology.**

☐ I **consent** to the voice recording of the interview.

Signature
Print name
Place and date
