# Supplementary material for: Perceptions of Health Care Professionals on the Integration and Use of AI in Clinical Cancer Care: Interview Study
Source: JMIR Hum Factors. 2026 Apr 20;13:e83240. doi: 10.2196/83240 (PMC13094801; doi:10.2196/83240)
Supplement: Multimedia Appendix 6 [file humanfactors-v13-e83240-s006.docx]

*Table A3: Themes, sub-themes, and associated codes*

| **Main theme** | **Sub-theme** | **Participants** | **Codes** |
| --- | --- | --- | --- |
| **General AI adoption perceptions (G)** | G1. AI developments | 9 (P1, P3, P7, P8, P9, P11, P13, P14, P16) | 10 |
|  | G2. Limitations & complexity | 3 (P3, P5, P13) | 5 |
|  | G3. AI applications | 9 (P1, P2, P3, P4, P5, P10, P11, P12, P13) | 16 |
| **Social Subsystem - Organization (SO)** | SO1. Organization-specific challenges to adopting AI | 5 (P1, P8, P9, P15, P18) | 14 |
|  | SO2. Facilitating factors at the organization level | 9 (P1, P3, P6, P8, P9, P11, P13, P15, P17) | 14 |
| **Social Subsystem – People (SP)** | SP1. Need for AI awareness | 9 (P3, P5, P6, P9, P10, P11, P12, P18, P19) | 12 |
|  | SP2. Attitudes towards AI | 13 (P1, P3, P5, P6, P8, P9, P10, P11, P13, P14, P15, P17, P19) | 22 |
|  | SP3. Age-related perceptions | 3 (P1, P7, P12) | 3 |
|  | SP4. Trust | 7 (P1, P4, P7, P8, P12, P17, P19) | 10 |
|  | SP5. Involvement in shaping the solution | 12 (P1, P2, P3, P4, P5, P6, P7, P10, P11, P12, P15, P17) | 23 |
|  | SP6. Responsibility and Accountability | 9 (P5, P7, P8, P10, P14, P15, P16, P17, P19) | 11 |
|  | SP7. Automation bias | 4 (P14, P15, P16, P19) | 5 |
|  | SP8. Clinician autonomy, behavioral impacts, and interpersonal factors | 7 (P3, P4, P7, P11, P12, P15, P19) | 13 |
|  | SP9. Effect on jobs, skills, and competencies | 16 (P1, P2, P3, P4, P5, P6, P7, P9, P10, P12, P13, P14, P16, P17, P18, P19) | 41 |
| **Technical subsystem (T)** | T1. AI potential | 10 (P3, P4, P6, P8, P9, P11, P12, P13, P15, P18) | 23 |
|  | T2. Fit-for-task design | 5 (P1, P2, P7, P9, P19) | 5 |
|  | T3. Ease of use | 7 (P1, P2, P3, P6, P9, P17, P19) | 14 |
|  | T4. Challenges related to data | 7 (P1, P5, P6, P7, P9, P10, P11) | 8 |
|  | T5. Interpretability and Explainability of AI tools | 10 (P3, P4, P5, P7, P10, P12, P13, P14, P17, P19) | 13 |
|  | T6. Unsuccessful AI efforts | 3 (P5, P6, P15) | 7 |
|  | T7. Effects of AI tools on workflow | 9 (P1, P2, P3, P9, P12, P14, P16, P17, P19) | 20 |
| **Impacts of AI Integration as indicators of joint optimization (J)** | J1. Benefits to clinical practice | 14 (P1, P2, P3, P4, P6, P7, P9, P11, P12, P13, P15, P16, P17, P19) | 40 |
|  | J2. Clinical benefits | 8 (P2, P6, P9, P10, P13, P16, P17, P19) | 16 |
|  | J3. Continuity | 8 (P1, P3, P6, P9, P10, P16, P17, P19) | 14 |
| **External systems (E)** | E1. Al and clinical guidelines | 7 (P4, P5, P6, P10, P15, P17, P19) | 9 |
|  | E2. Macro-level enablers | 4 (P2, P6, P9, P12) | 4 |
|  | E3. Vendor stability | 1 (P1) | 1 |
|  | E4. Regulatory influence | 4 (P3, P7, P9, P10) | 5 |
